# Are digital health interventions valuable to support patients with cancer and caregivers? An umbrella review of web‐based and app‐based supportive care interventions

**DOI:** 10.1002/cam4.6695

**Published:** 2023-11-08

**Authors:** Valentyn Fournier, Christelle Duprez, Delphine Grynberg, Pascal Antoine, Kristopher Lamore

**Affiliations:** ^1^ Universite de Lille, CNRS, UMR 9193—SCALab—Sciences Cognitives et Sciences Affectives Lille France; ^2^ Institut Universitaire de France Paris France

**Keywords:** digital intervention, patients, relatives, supportive care, umbrella review

## Abstract

**Background:**

Digital health technologies have expanded tremendously in the last two decades, creating an emerging research and clinical field. They are regarded as cost‐effective, and their use in healthcare is prioritized by many countries. However, the constant evolution of these technologies has led to an abundance of related literature. Thus, we conducted an umbrella review to identify and characterize digital supportive care interventions for patients with cancer and their relatives.

**Methods:**

A preregistered umbrella review was conducted (PROSPERO registration number CRD42022333110). Five databases were searched (Embase, PsycINFO, PubMed, CINAHL, and the Cochrane Library). To be considered, studies had to be systematic reviews or meta‐analyses, be performed on pediatric or adult patients with cancer or survivors or their relatives, report results on web‐based or app‐based supportive care interventions, and measure psychological, functional, or behavioral variables or quality of life related to cancer. The methodological quality of the studies was assessed using the AMSTAR‐2 tool.

**Findings:**

Twenty eligible studies were identified. Most of the included studies reported results from adult patients with cancer. Globally, digital interventions were shown to be effective for physical activity in patients with cancer but had mixed results regarding emotional outcomes and quality of life. Additionally, a lack of methodological quality was noted for most of the included reviews.

**Discussion:**

Digital supportive care interventions could be an effective tool in cancer care for some outcomes. Recommendations have been formulated for further research in this field using adapted methodologies for the development of digital health interventions.

## 1 INTRODUCTION

The global burden of cancer is growing and improvements in clinical care are urgently needed to face it.^1^, ^2^, ^3^ The improvements in cancer care require more healthcare professionals to meet basic clinical demand in oncology units^4^ and in supportive care units that are already understaffed.^5^ There is a need for innovative, cost‐effective approaches for optimal patient management, and psychosocial care that do not require workforce increases.^6^, ^7^, ^8^ In some cases, new effective psychosocial face‐to‐face interventions (e.g., group interventions, mindfulness) can be more expensive that institutions cannot afford.^8^ These costs can be related, for example, to the professional who takes more time with one patient to the detriment of others, additional training costs, or increased demand. In addition, given the high risk of psychopathological disorders in the relatives of patients with cancer, such new cost‐effective interventions should also be available to them.^9^

In the last two decades, digital technologies have expanded tremendously. Technologies developed to support human health and well‐being are more recent, and their effectiveness and impact on the healthcare system need to be evaluated constantly. Digital technologies comprise electronic health (eHealth), mobile health (mHealth), telemedicine, telemonitoring, and digital therapeutics. Digital health interventions can be used to promote healthy behaviors, support individuals with mental health conditions or long‐term conditions such as cancer, and facilitate care pathway.^10^ Additionally, these interventions can facilitate care access for underserved groups and maintain patient‐centered care within a system involving family members in care.^11^ National and international guidelines outline the importance of implementing health‐related digital technologies to support care.^12^ However, despite the growing interest reflected in the numerous extant systematic reviews and meta‐analyses of digital interventions in cancer care, the collected evidenced‐based results need to be synthesized to confirm the relevance and efficacy of digital health interventions in providing psychosocial support to patients with cancer and their relatives.

Thus, we conducted an umbrella review aiming to identify the existing digital interventions developed to provide supportive care to patients with cancer and their relatives in the cancer care continuum. The secondary aim was to report how those interventions influence outcomes of interest.

## 2 METHODS

This umbrella review was preregistered on PROSPERO (International prospective register of systematic reviews, CRD42022333110). It adheres to the Preferred Reporting Items for Systematic Reviews and Meta‐Analyses (PRISMA) guidelines^13^ and follows the recommendations of Aromataris et al.^14^

### 2.1 Search strategy

Five databases were searched on April 4, 2022 (and rerun on November 23, 2022): Embase, PsycINFO, PubMed, CINAHL, and the Cochrane Library. Gray literature (Google Scholar) and references included in studies were checked to ensure a comprehensive search. Searches were performed using a comprehensive list of keywords related to the type of article (systematic reviews and meta‐analyses), digital interventions, and cancer (see Appendix 1).

### 2.2 Study selection

Study selection was performed with the web application Covidence (Covidence systematic review software, Veritas Health Innovation, Melbourne, Australia. Available at www.covidence.org). All the steps were performed independently by two authors (VF and KL). After removal of duplicates, the titles and abstracts of the studies were screened. Then, a full text review of the remaining studies was performed. In case of disagreement, conflicts were resolved through discussion about the motivation for the choice until an agreement was reached. Finally, data extraction and summarization were performed. An inter‐rater agreement rate was calculated for each stage of the process.

### 2.3 Inclusion criteria

To be included, the systematic reviews or meta‐analyses had to meet each of the following PICOS criteria.^15^, ^16^ The population of interest comprised patients with cancer regardless of age and/or their relatives throughout the cancer continuum, from diagnosis to survivorship (Population). Interventions had to be either web‐based or app‐based digital health psychosocial, behavioral, or supportive care interventions. For this review, given the scarcity of other interventions (e.g., telemedicine, telemonitoring), the considered digital interventions included web‐based interventions and app‐based interventions (Intervention). Where applicable, the comparator had to be a usual care group or a group of participants exposed to another intervention (Comparator). The outcomes of interest included psychological variables (e.g., anxiety, depression), functional variables (e.g., pain, cognitive functioning), behavioral variables (e.g., physical activity), or the quality of life related to the cancer (Outcome). Only systematic reviews of quantitative studies and meta‐analyses were included (Study type).

The abstracts and full text had to be written in English, French, or Spanish. Only studies published after 2000 were considered.

### 2.4 Exclusion criteria

Items were excluded if (i) they did not exclusively consider patients with cancer or their relatives, (ii) they did not mainly relate to web‐based or app‐based digital interventions, (iii) the intervention was exclusively implemented via social media, or (iv) they reviewed case reports, observational studies, qualitative studies, or study protocols.

### 2.5 Study quality assessment

The quality of the reviews or meta‐analyses was assessed independently by VF and KL using the Assessing the Methodological quality of Systematic Reviews tool (AMSTAR‐2).^17^ AMSTAR‐2 consists of 16 items (14 for systematic reviews and meta‐analyses and two additional only for meta‐analyses).

### 2.6 Data extraction and synthesis

Data were extracted independently by VF and KL (see Appendix 2). The two respective versions were compared and discussed in case of disagreement. Results regarding web‐based and mobile‐based interventions were distinguished because of potential differences due to the medium.

According to recommendations, a narrative synthesis of the data was performed distinguishing intervention type, aim of the intervention, and outcomes.^14^

### 2.7 Role of the funding source

The funding source had no role in study design, data collection, data analysis, data interpretation, writing of the report, or in the decision to submit the paper for publication.

## 3 RESULTS

**FIGURE 1:**
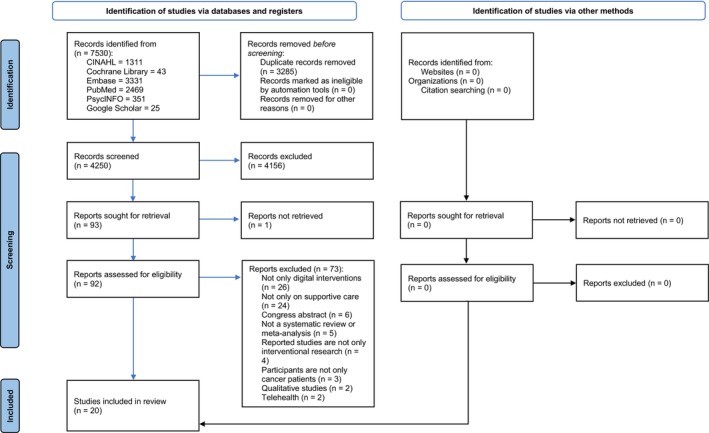
PRISMA flowchart.

Three thousand eight hundred and twenty articles after primary systematic search and were screened based on their titles and abstracts (inter‐rater agreement: 98.53%). As a result, 83 articles were chosen for full‐text review (inter‐rater agreement: 72.29%; see Appendix 3) and 18 fully met the inclusion criteria, reporting on a total of 255 original studies after the removal of duplicates (see Appendix 4). The rerun of the systematic search led to the identification of 394 supplementary articles leading to the identification of two articles after applying same screening process. Finally, 20 reviews were included in this umbrella review (see Figure 1).

### 3.1 Studies characteristics

Eligible reviews were published between 2015 and 2022. Sixteen reported results from adult patients with cancer undergoing active treatment (diverse localizations^18^, ^19^, ^20^, ^21^, ^22^, ^23^, ^24^, ^25^, ^26^ and breast cancer^27^, ^28^), patients with an advanced stage of the disease,^29^ or cancer survivors^30^, ^31^, ^32^, ^33^; two reviews were related to pediatric cancers (diverse cancer localizations for patients under active treatment and survivors^34^ or survivors only^35^); and two dealt with the caregivers of patients with cancer (one with both adult cancer survivors and their partners^36^ and one with caregivers only^37^) (see Appendix 4).

The quality assessment (see Table 1) showed discrepancies between the reviews. Eleven reviews fulfilled at least half of the criteria,^18^, ^22^, ^24^, ^25^, ^27^, ^28^, ^29^, ^30^, ^32^, ^34^, ^35^ whereas nine did not.^19^, ^20^, ^21^, ^23^, ^26^, ^31^, ^33^, ^36^, ^37^ (inter‐rater agreement: 93.66%). Overall ratings of confidence were applied to the reviews.^20^ Therefore, the rating of confidence was high for three reviews,^24^, ^27^, ^34^ moderate for 2,^25^, ^28^ low for 3,^18^, ^22^, ^35^ and critically low for 12.^19^, ^20^, ^21^, ^23^, ^26^, ^29^, ^30^, ^31^, ^32^, ^33^, ^36^, ^37^

**TABLE 1:** Appraisal of the quality of studies (AMSTAR‐2) (colors needed).

	Buneviciene, 2021	Dorri, 2020	Ester, 2021	Goliță, 2019	Haberlin, 2018	Hong, 2021	Huang, 2020	Kaltenbaugh, 2015	Kamalumpundi, 2022	Kang, 2018	Kim, 2019	Kiss, 2019	McAlpine, 2015	Qan'ir, 2019	Ramsey, 2020	Seiler, 2017	Singleton, 2022	Wan, 2022	Zhang, 2022	Zheng, 2020
Item 1																				
Item 2																				
Item 3																				
Item 4																				
Item 5																				
Item 6																				
Item 7																				
Item 8																				
Item 9 RCTs																				
Item 9 NRSI				*			*			*		*					*		*	
Item 10																				
Item 11 RCTs		**	**	**	**			**		**	**	**	**	**	**					
Item 11 NRSI		**	**	**	**		*	**		**	**	**	**	**	**		*		*	*
Item 12		**	**	**	**			**		**	**	**	**	**	**					
Item 13																				
Item 14																				
Item 15		**	**	**	**			**		**	**	**	**	**	**					
Item 16																				

*Note*: 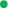, criteria met; 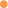, criteria partially met; 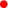, criteria not met or unable to answer from the available information; *, includes only RCTs; **, no meta‐analysis was conducted.

Abbreviations: RCTs, randomized controlled trials; NRSI, non‐randomized studies of interventions

### 3.2 Types of interventions

Of the 20 reviews, 12 reported results from both web‐based and app‐based interventions,^18^, ^20^, ^22^, ^24^, ^27^, ^28^, ^29^, ^30^, ^32^, ^33^, ^34^, ^35^ seven from web‐based interventions only,^19^, ^23^, ^25^, ^26^, ^31^, ^36^, ^37^ and one from app‐based interventions only.^21^

Two reviews did not specify the duration of the interventions,^21^, ^37^ one did for only one of the interventions (six weeks^20^), and one was not clear on this point (potentially 6–12 weeks^34^). The remaining reviews reported interventions that were between 1 week and 52 weeks long.

### 3.3 Summary of evidence and effectiveness of the interventions

The reported interventions were considered effective when at least half of them showed positive results for a considered variable (for narrative synthesis) or when meta‐analysis showed significant results. The global effect of the interventions and the parts of primary studies showing a positive effect are depicted in Tables 2, 3, and 4. Neither statistical pooling of the results nor a meta‐analysis was performed because of the high heterogeneity of the included reviews.

**TABLE 2:** Summary of evidence for web‐based interventions.

Category of intervention	Target of the intervention	Articles (first author)	Outcome of interest	Number of studies
Emotions and emotional disorders	Anxiety	Kamalumpundi, 2022 Ramsey, 2020	Anxiety	13
	Depression	Kamalumpundi, 2022 Ramsey, 2020	Depression	12
	Post‐traumatic stress	Ramsey, 2020	Post‐traumatic stress	9
	Psychological distress	Kamalumpundi, 2022	Psychological distress	9
	Cognitive behavioral therapy	McAlpine, 2015 Qan'ir, 2019	Anxiety	5
		McAlpine, 2015 Qan'ir, 2019 Seiler, 2017	Depression	7
		Goliță, 2019	Emotional distress	12
		Buneviciene, 2021	Emotional well‐being	2
		Seiler, 2017	Fatigue	4
		Qan'ir, 2019 Seiler, 2017 Buneviciene, 2021 Goliță, 2019 McAlpine, 2015	HRQoL	17
		Seiler, 2017	Insomnia	1
		Buneviciene, 2021	Physical health	1
		McAlpine, 2015	Sleep quality	1
		Buneviciene, 2021	Social functioning	1
	Mindfulness/stress	Seiler, 2017	Fatigue	1
		Buneviciene, 2021	HRQoL	3
		Goliță, 2019	Emotional distress	8
	Coping	McAlpine, 2015	Emotional well‐being	1
		McAlpine, 2015	HRQoL	1
	Interactive support	Buneviciene, 2021	HRQoL	1
		Goliță, 2019	Emotional distress	8
		Goliță, 2019	HRQoL	2
Behaviors	Binge drinking	Ramsey, 2020	Binge drinking	1
	Smoking cessation	Ramsey, 2020	Smoking cessation	2
	Nutrition and physical activity	Kiss, 2019	Anxiety	1
		Kiss, 2019	Diet	3
		Kiss, 2019	Fatigue	3
		Kiss, 2019	HRQoL subdimensions	3
		Kiss, 2019	Insomnia	1
		Kiss, 2019	Mental health	1
		Kiss, 2019	Physical activity	5
		Kiss, 2019	Pain	2
	Physical activity	Buneviciene, 2021	Emotional well‐being	1
		Seiler, 2017	Fatigue	1
		Buneviciene, 2021	HRQoL	2
		Dorri, 2019 Ester, 2021 Haberlin, 2018 Ramsey, 2020	Physical activity	57
	Weight management	Buneviciene, 2021	HRQoL	1
Information and self‐management	Cancer knowledge	Ramsey, 2020	Cancer knowledge	1
	Information/psychoeducation	McAlpine, 2015 Qan'ir, 2019	Anxiety	2
		McAlpine, 2015 Qan'ir, 2019	Depression	2
		McAlpine, 2015	Fatigue	1
		McAlpine, 2015 Buneviciene, 2021 Qan'ir, 2019	HRQoL	5
		McAlpine, 2015	Physical activity	1
		Qan'ir, 2019	HRQoL	3
	Self‐management	Seiler, 2017	Fatigue	2
		Seiler, 2017	Fatigue self‐efficacy	1
		Seiler, 2017	Insomnia	1
		Hong, 2021	Binge drinking	1
		Hong, 2021	Fitness outcomes	2
		Hong, 2021	Working memory	1
		Seiler, 2017 Hong, 2021	Physical activity	3
Cognitive function	Cognitive function	Kim, 2019	Anxiety	3
		Kim, 2019 Seiler, 2017	Cognitive function (several outcomes)	4
		Kim, 2019	Depression	3
		Kim, 2019 Seiler, 2017	Fatigue	3
		Seiler, 2017	Global health status	1
		Kim, 2019	HRQoL	2
		Kim, 2019	Stress	2
Sexual function	Sexuality	Kang, 2018	Dyadic functioning	1
		Kang, 2018	Partner sexual function	2
		Kang, 2018	Patient sexual function	3
		Kang, 2018	Psychological distress	1
		Kang, 2018	HRQoL	1
Multitargeted or nonspecific interventions	Web‐based (outcome not clear)	Zhang, 2022	Depression	6
		Zhang, 2022	HRQoL	5
		Zhang, 2022	Self‐efficacy	3
		Zhang, 2022	Symptom distress	3
	Web‐based multi‐component intervention	Kaltenbaugh, 2015	Physical activity	1
		Kaltenbaugh, 2015	Psychological variables	5
		Kaltenbaugh, 2015	Social support	1
		Wan, 2022	Self‐efficacy	7
		Wan, 2022	Anxiety	3
		Wan, 2022	Depression	8
		Wan, 2022	HRQoL	3
		Wan, 2022	Psychological distress	5
		Wan, 2022	Cancer‐specific psychological distress	4
		Huang, 2020	Anxiety	2
		Huang, 2020	Depression	2
		Huang, 2020	Fatigue	13
		Huang, 2020	HRQoL	2
		Huang, 2020	Sleep quality	2

Note: 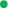, global efficacy; 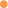, not significant; 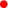, deleterious.

Abbreviations: HRQoL, health‐related quality of life.

**TABLE 3:** Summary of evidence for app‐based interventions (colors needed).

Category of intervention	Target of the intervention	Articles (first author)	Outcome of interest	Number of studies
Emotions and emotional disorders	Anxiety	Kamalumpundi, 2022	Anxiety	3
	Depression	Kamalumpundi, 2022	Depression	3
	Cognitive behavioral therapy	Buneviciene, 2021	HRQoL	3
	Mindfulness/stress	Buneviciene, 2021	QoL	2
	Social support	Buneviciene, 2021	HRQoL	2
Behaviors	Physical activity	Buneviciene, 2021	HRQoL	5
		Buneviciene, 2021	Social functioning	1
		Dorri, 2019	Physical activity	26
	Nutrition and physical activity	Kiss, 2019	Physical activity	3
		Kiss, 2019	HRQoL	1
Information and self‐management	Information/psychoeducation	Buneviciene, 2021	HRQoL	1
	Digital self‐management interventions	Hong, 2021	Physical activity	2
		Hong, 2021	HRQoL	1
		Hong, 2021	Social functioning	1
Symptoms	Pain	Ramsey, 2020	Pain	1
		Zheng, 2020	Anxiety	5
		Zheng, 2020	Fatigue	1
		Zheng, 2020	Pain/pain catastrophizing	9
		Zheng, 2020	Pain self‐efficacy	1
		Zheng, 2020	HRQoL	5
		Zheng, 2020	Symptom reporting	2
Nonspecific interventions	Mobile app	Zhang, 2022	HRQoL	1
	Mobile app with instant messaging module	Zheng, 2020	Pain	3

*Note*: 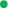, global efficacy; 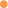, not significant; 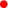, deleterious.

Abbreviations: HRQoL, health‐related quality of life.

**TABLE 4:** Summary of evidence for both web‐based and app‐based interventions (colors needed).

Category of intervention	Target of the intervention	Articles (first author)	Outcome of interest	Number of studies
Behaviors	Physical activity	Haberlin, 2018	Physical activity	4
Multitargeted or nonspecific interventions	Multi‐component intervention	Singleton, 2022	HRQoL	11
		Singleton, 2022	Anxiety	6
		Singleton, 2022	Depression	6
		Singleton, 2022	Psychological distress	3
		Singleton, 2022	Self‐efficacy	7
		Singleton, 2022	Fatigue	5

*Note*: 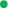, global efficacy; 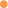, not significant.

Abbreviations: HRQoL, health‐related quality of life.

### 3.4 Web‐based interventions

#### 3.4.1 Interventions on emotions and emotional disorders

Interventions respectively targeting anxiety and depression showed no significant efficiency in either adult patients or pediatric patients (10 studies on anxiety and 14 on depression).^29^ However, when the target was unspecified psychological distress, the interventions seemed to be moderately efficient (nine studies).^29^

Some reviews reported results from interventions using cognitive behavioral therapy (CBT), involving various elements of this approach (e.g., cognitive reframing, coping skills training, cognitive restructuration). Web‐based CBT interventions showed positive effects on emotional distress (12 studies),^26^ fatigue (four studies),^30^ emotional well‐being (two studies),^33^ insomnia (one study),^30^ sleep quality (one study),^23^ and social functioning (one study).^33^ However, these interventions showed no significant results regarding Health‐Related Quality of Life (HRQoL; 17 studies),^18^, ^23^, ^26^, ^30^, ^33^ depression (seven studies),^18^, ^23^, ^30^ anxiety (five studies),^18^, ^23^ or physical health (one study).^33^ Other interventions targeting mindfulness to reduce stress in adult patients showed a positive effect on emotional distress (eight studies),^26^ HRQoL (three studies), ^33^ and fatigue (one study).^30^ Interventions targeting coping skills showed mixed results, with a positive effect on emotional well‐being (one study) but no significant effect on HRQoL (one study).^23^

#### 3.4.2 Interventions on behaviors

Physical activity and nutrition‐related behaviors were the main behavioral targets of web‐based interventions. Physical activity interventions were shown to be efficient for enhancing physical activity (19 studies),^24^, ^28^, ^32^ HRQoL (two studies),^33^ and emotional well‐being (one study).^33^ However, no significant effect was observed for fatigue (one study).^30^

When nutrition and physical activity were considered together,^23^ interventions were efficient for fatigue (three studies), HRQoL (three studies), and insomnia (one study) but not for physical activity amount (five studies), diet (three studies), pain (two studies), or anxiety (one study). A study showed no effect of the intervention on the experimental group but an improvement of mental health in the waitlisted control group. Interventions targeting weight management had no effect on HRQoL (one study).^33^

#### 3.4.3 Interventions on information and self‐management

Interventions targeting knowledge had a positive impact on fatigue (13 studies), depression (two studies), HRQoL (two studies), and sleep quality (two studies) but not on anxiety (two studies).^19^ When interventions aimed for psychoeducation, only an improvement in fatigue symptoms was observed (one study),^23^ whereas no effect was observed for anxiety (two studies), depression (two studies),^18^, ^23^ HRQoL (five studies),^18^, ^23^, ^33^ or physical activity (one study).^23^

One review reported the effectiveness of self‐management interventions in adult patients with cancer.^21^ These interventions had a positive effect on fatigue self‐efficacy (one study) and insomnia (one study). However, they had no significant effect on fatigue (two studies) or physical activity (two studies).

#### 3.4.4 Interventions on cognitive function

Cognitive function‐specific interventions were shown to be efficient regarding cognitive functions (four studies), fatigue (three studies),^30^, ^31^ and global health status (one study).^30^ They had no significant effect on emotional outcomes, such as anxiety (three studies), depression (three studies), or stress (two studies), or HRQoL (two studies).^31^

#### 3.4.5 Interventions on sexual function

Only one review synthesized the results from studies on sexual function using a dyadic approach.^36^ Online interventions were efficient regarding both patients' and partners' sexual function (three and two studies, respectively), unspecified psychological distress (one study), and HRQoL (one study) but not dyadic functioning (one study).

#### 3.4.6 Multitargeted or nonspecific interventions

Some reviews did not specify the target of the reviewed intervention, forcing a global interpretation of their results. In this context, multitargeted web‐based interventions were shown to be efficient in adult patients regarding emotional disorders, including depression (14 studies),^20^, ^25^ general and cancer‐specific psychological distress (five and four studies, respectively), anxiety (three studies),^25^ and symptom distress (three studies),^20^ self‐efficacy (10 studies), and HRQoL (eight studies).^20^, ^25^ A review on caregivers^37^ showed efficiency regarding psychological variables (five studies), no significance regarding physical burden (one study), and a deleterious effect of interventions for perceived social support (one study).

#### 3.4.7 Interventions in pediatric patients or childhood cancer survivors

In pediatric patients and childhood cancer survivors, a review suggested that web‐based interventions were efficient for physical activity (eight studies) improving depression (two studies) but not anxiety (three studies).^34^ Interventions targeted at binge drinking and smoking cessation were shown to have a positive effect on the former but no significant effect on the latter (one and two studies, respectively).^34^ When they targeted knowledge about cancer, interventions had no significant effect in pediatric patients and childhood cancer survivors (one study).^34^ One study reviewed the effectiveness of self‐management interventions in pediatric patients and childhood cancer survivors.^35^ These interventions had a positive effect on binge drinking (one study) and working memory (one study) (14). However, they had no significant effect on physical activity (one study).^35^

### 3.5 App‐based interventions

#### 3.5.1 Interventions on emotions and emotional disorders

Interventions targeting anxiety and depression were effective for both (three studies each).^29^ App‐based CBT interventions did not have a significant effect on HRQoL (three studies), whereas mindfulness interventions did (two studies).^33^

Studies targeting perceived social support to cope with illness showed no significant results regarding HRQoL (two studies).^33^

#### 3.5.2 Interventions on behaviors

Physical activity‐targeting app‐based interventions were efficient in enhancing physical activity (26 studies)^28^ and HRQoL (five studies).^33^ A single study showed a detrimental effect of these interventions on social functioning.^33^ Interventions targeting both nutrition and physical activity showed no significant effect on physical activity amount (three studies) or HRQoL (one study).^22^

#### 3.5.3 Interventions on information and self‐management

App‐based psychoeducational interventions did not have any significant effect on HRQoL in adult patients with cancer (one study).^33^

#### 3.5.4 Interventions on pain symptoms

Interventions targeting pain as a symptom were efficient against pain catastrophizing (nine studies), anxiety (five studies), and fatigue (one study) and for enhancing HRQoL (five studies).^21^ These interventions had deleterious effects on pain self‐efficacy according to one study.^21^ When compared, mobile‐based interventions using an instant messaging module in adult patients (three studies) seemed more efficient against pain symptoms than interventions without such a module in pediatric patients (one study).^21^, ^34^ More generic symptom‐reporting applications had a positive effect on HRQoL (one study).^18^

#### 3.5.5 Interventions in pediatric patients or childhood cancer survivors

Digital self‐management interventions in childhood cancer survivors had positive effects on social functioning (one study) but no significant effect on physical activity (two studies) or HRQoL (one study).^35^

### 3.6 Unspecified interventions

An included review did not allow for distinguishing web‐based from app‐based interventions.^27^ These interventions were shown to have a positive effect on HRQoL (11 studies), self‐efficacy (seven studies), fatigue (five studies), and unspecified psychological distress (three studies) but not anxiety (six studies) or depression (six studies).

Another review did not allow for distinguishing the format of physical activity‐targeting interventions and showed no significant results regarding physical activity (four studies).^32^

## 4 DISCUSSION

This umbrella review highlighted that most studies referred to adult patients with cancer or survivors, with only two on pediatric patients or survivors and two on caregivers of patients. Digital interventions were shown to be effective for physical activity but produced mixed results regarding emotional outcomes (depending on their nature, i.e., anxiodepressive symptoms or unspecified psychological distress). However, the quality assessment of the included reviews demonstrated that most of them suffered from a lack a methodological quality.^17^

The two main categories of outcomes were emotional (mainly anxiety, depression, and emotional distress) and behavioral (mostly physical activity). Digital interventions showed mixed results for emotional variables. Even if some studies tended to show a positive effect of these interventions on anxiety (app‐based interventions^21^, ^29^) and depression (web‐based^19^, ^20^, ^25^, ^30^, ^34^ and app‐based^29^ interventions), the general tendency was to not observe statistically significant effects.^18^, ^19^, ^23^, ^27^, ^29^, ^31^, ^34^ However, regarding psychological distress, studies showed positive effects for both web‐based and app‐based interventions.^25^, ^26^, ^27^, ^29^, ^36^ The discrepancy in the results between anxiety and depression on one hand and unspecified psychological distress on the other may be surprising given the conceptual proximity of those two categories of variables. This could be explained by the fact that emotional distress and emotional well‐being are a blurry concept in digital health research, which may contain anxio‐depressive symptoms.^38^ This confusion could lead to inconsistencies in the way these concepts are measured, leading to discrepancies in the results.

Regarding physical activity, the observed results were positive whether interventions were web‐based or app‐based.^22^, ^23^, ^24^, ^28^, ^30^, ^32^, ^34^, ^35^ Physical activity was the most frequently measured outcome in the studies. One original study reported by two reviews described positive effects on binge drinking.^34^, ^35^ However, other behavioral outcomes (i.e., diet, smoking cessation) seemed not to be significantly modified by digital interventions.^22^, ^34^

On a functional level, the results of the interventions revealed globally good outcomes for some variables (e.g., fatigue,^19^, ^22^, ^23^, ^30^, ^31^ sleep^19^, ^22^, ^23^, ^30^) but nonsignificant effects for others (e.g., pain^21^, ^22^, ^34^). When HRQoL was considered alone, app‐based interventions^18^, ^21^, ^22^, ^27^, ^33^, ^34^ were shown to be more effective than web‐based ones.^18^, ^20^, ^23^, ^25^, ^26^, ^30^, ^31^, ^33^, ^36^ However, it seems important to consider the variability in the conceptualization and measurement of HRQoL. Similarly, another element that could explain the variability in effectiveness regarding this variable is the targeted outcome of the interventions. For example, pain‐targeted interventions or more generally functional interventions tended to be more effective regarding HRQoL than others. In this sense, it could be relevant to consider some variables more as mediators of the efficacy of the intervention than as final outcomes.

### 4.1 Perspectives and recommendations

Some studies suggested nonsignificant effects on some outcomes (e.g., anxiety, depression). This result could be due to the declared target of the intervention that may differ from the outcome considered (e.g., an intervention developed to enhance daily physical activity but for which emotional variables are evaluated as a primary outcome). In this context, it might be crucial to correctly target the outcomes evaluated as they might affect the interpretation of the real efficacy of the intervention. To do so, it appears essential to clearly identify the pathways by which the interventions could determine the outcomes of interest.^39^ This requires modeling of the supposed action mechanism of the interventions and thus, a solid theoretical background in their conception.^40^

Constating the mitigated efficacy of the interventions, it is possible to wonder if the measurements are being limited. Indeed, although some considered baseline levels of outcomes, others did not. Thus, the absence of a significant effect could be due to the fact that some patients don't show a pathological state at the beginning of the intervention and, therefore, don't benefit from the intervention. For these reasons, some nonsignificant results should be interpreted cautiously, and further research should be conducted that matches the target of the interventions and the identified outcomes, and measures the baseline levels of outcomes of interest to avoid a floor effect. Implementing a baseline measure could also help develop adaptive interventions that would target outcomes for which a pathological level is observed at the beginning of the intervention. In the same vein, interventions could also continuously monitor the outcomes to adapt during their use. To do so, a strong call for the development and systematic use of standardized and sensitive measurement tools should be made (e.g., by implementing ecological momentary assessments).^41^

Adherence to digital interventions constitutes a common but complex problem that should be considered because of its potential influence on effectiveness.^42^, ^43^ A recent scoping review found that individual characteristics can influence adherence to digital interventions and thus, their effectiveness.^44^ Beyond studying the effects of an intervention using patient‐reported outcomes (PROs) as patient‐centered evaluation tools,^45^ it appears that measuring user adherence and perceived obstacles to use (e.g., numerical literacy, preference for computers or smartphones) as well as their acceptability,^43^ is pertinent to improve the development of interventions. However, identifying these factors at the end of the development process could be more costly. Thus, it seems wise to investigate these parameters at the beginning of the development phase. Building interventions with patients based on their needs and characteristics seems necessary, as their experiential knowledge is important for intervention design.^46^, ^47^, ^48^ Moreover, involving patients from the early stages of development could help to address some concerns regarding the preservation or exacerbation of social inequalities in access to devices, bringing an in‐context point‐of‐view. Indeed, it has been observed that some patients could find it difficult to adopt the interventions because they lack access to the digital technology or because of socioeconomic deprivation.^49^ This represents a problem, given that a chain reaction could lead to significant socioeconomic inequalities.^50^ For those reasons, potential socioeconomic inequalities in implementing and adopting of digital interventions should be a crucial interest for the development of future interventions, as well as their cost‐effectiveness. To date, these aspects have rarely been investigated and reported in publications.

A large proportion of the included reviews selected studies that used an RCT design to investigate the effects of interventions. Although this design is widely used in studies of pharmacological interventions and considered the gold standard,^51^ it has been criticized in the context of non‐pharmacological interventions such as digital interventions.^43^ RCTs are adopted only when the studied intervention fulfills certain requirements (i.e., stability in providing the intervention, fidelity of the intervention, and likelihood of clinical significance of the benefits of the intervention).^52^ Given the characteristics of digital interventions, which can be rather complex, a model shift in the studies evaluating their effects could be useful. In this regard, Skivington et al. suggested specifying the perspective from which the intervention is evaluated (i.e., efficacy, effectiveness, theory‐based, systems). This classification calls into question the setting (i.e., ideal, experimental, real‐world) of the intervention, since RCTs are designed for ideal or experimental settings but digital interventions are characterized by the variability of their settings. Beyond the methodological considerations in the conception of studies, some authors claim that a move from RCTs to other, more individualized research paradigms would bring changes in data analysis, causing a shift from sample analysis to individual analysis (e.g., N‐of‐1 studies).^53^ For these reasons, a shift in research methods might enable more consistency in evaluation and greater reliability in the results of the studies.^51^

The included reviews were characterized by their lack of methodological quality, measured using AMSTAR‐2.^17^ One of the most significant factors contributing to quality impairment was the lack of descriptions of the included interventions, which prevents global interpretation regarding the elements that have the greatest effect on a specific outcome. Thus, adopting strict transparency in studies and their reporting is strongly recommended. This could be achieved in several ways. For example, more open practices in research could integrate systematic preregistration, registered reports, data sharing plans, the dissemination of reproducible analysis code or detailed intervention contents, preprints, or data sharing.^54^, ^55^ Among other open science principles, the preregistration of interventional studies could help buid greater trust in interventional research, reducing the potential for a lack of transparency, selective reports in the results, or false‐positives.^55^, ^56^ Methodological quality evaluation tools also could be improved by implementing items on transparency in the description of interventions and their results.^56^ Other factors that could benefit from greater transparency include the level of adherence and the attrition rate and their determinants. Despite being common in interventional studies, few studies report these items.^57^ Applying open science principles to interventional research is not only crucial for better reproducibility but would also enable better implementation of the interventions in the real world, allowing them to benefit more people and increasing their benefit–cost ratio.^58^

The recommendations formulated are summarized in Box 1. Detailed recommendations to report psychosocial trials exist, such as the CONSORT statement,^59^ GUIDED^60^ or TIDieR^61^ checklists. However, these recommendations do not seem to be known or used by some in the scientific committee and can also be updated to include new elements, as discussed above.

BOX 1 **Recommendations for future interventional study designs**

**Table:** 

Refer to a concrete theoretical anchoring to identify the mechanisms of action of the intervention
Consider patients in the development of interventions, their perceived obstacles to utilization and potential inequality in implementation of the intervention
Implement a solid measurement protocol: from baseline, all along the pathway, with standardized and sensitive tools
Get out of the systematic use of randomized controlled trials model and look for alternative adapted evaluation models
Bring strict transparency in the development with pre‐registration or registered reports and data sharing plans
Systematically report and discuss attrition rates, level of adherence and satisfaction of patients
Assess the cost of the intervention for everyone (i.e., institutions, clinicians, and end‐users)

### 4.2 Limitations

The limitations of this umbrella review include the fact that concepts are generally poorly defined. Indeed, given that they encompass several types of interventions, digital interventions remain a relatively blurry concept in the literature.^62^, ^63^ Two issues stem from this poor definition. First, there may be confusion in the characterization of the interventions and interpretation of the results. Thus, it appears crucial to bring more rigor in description and definition of digital interventions in the context of the considerable digitalization of the healthcare.^64^ Regarding this definition, it appears that conclusions could be refined if some intervention components were clearly defined in reviews (e.g., CBT). Second, because of the heterogeneity in the methodologies, outcomes of interest, and methods of efficiency evaluation, it was impossible to conduct a quantitative synthesis. For this reason, it is impossible to systematically characterize the heterogeneity in the reviews as well as a potential publication bias. Third, most of the included reviews considered adult patients with cancer. Thus, although the objective of this umbrella review was to systematically synthesize the effectiveness of digital interventions in cancer care regarding both patients and their relatives, it remains impossible to draw solid conclusions for pediatric patients and caregivers. Finally, the recommendations for conducting umbrella reviews may lack flexibility in the case of interventional studies. It could be more informative to perform a rescreening of the primary studies to assess some of their aspects (e.g., contents of the interventions).

## 5 CONCLUSION

As medical care and therapeutics have allowed patients to live longer, it has become increasingly important to develop cost‐effective supportive care interventions for patients and their relatives. This umbrella review synthesized the results from 20 systematic reviews and meta‐analyses to establish the effectiveness of digital interventions in cancer. The evidence shows that interventions are numerous and globally efficient. However, great heterogeneity in the interventions is observed, and several reviews do not fulfill the methodological requirements of reporting results from interventional studies, leading to doubts about their conclusions. Further research is needed to develop interventions that are methodologically founded, allowing for scrupulous testing to determine what type of intervention is efficient and on what outcome. Additionally, clearer recommendations in intervention research and related publications are needed, as the existing ones are not comprehensive enough.

## AUTHOR CONTRIBUTIONS

**Valentyn Fournier:** Conceptualization (equal); data curation (equal); formal analysis (equal); investigation (equal); methodology (equal); project administration (equal); resources (equal); software (equal); validation (equal); visualization (equal); writing – original draft (equal); writing – review and editing (equal). **Christelle Duprez:** Conceptualization (equal); methodology (equal); validation (equal); visualization (equal); writing – review and editing (equal). **Delphine Grynberg:** Conceptualization (equal); methodology (equal); validation (equal); visualization (equal); writing – review and editing (equal). **Pascal Antoine:** Conceptualization (equal); methodology (equal); validation (equal); visualization (equal); writing – review and editing (equal). **Kristopher Lamore:** Conceptualization (equal); data curation (equal); formal analysis (equal); funding acquisition (equal); investigation (equal); methodology (equal); project administration (equal); resources (equal); software (equal); supervision (equal); validation (equal); visualization (equal); writing – original draft (equal); writing – review and editing (equal).

## FUNDING INFORMATION

This work was funded by the French National Cancer Institute (Institut National du Cancer, grant INCA/16136) in collaboration with the Université de Lille, the SCALab laboratory, the ONCOLille Institute, and the Centre Oscar Lambret that support the research chair opsyrii “innovations in psycho‐oncology and intervention research.”

## CONFLICT OF INTEREST STATEMENT

None.

## Supporting information


**Appendix 1.** Search strategies
**Appendix 2.** Characteristics of the studies
**Appendix 3.** Excluded full text
**Appendix 4.** Original studies

## Data Availability

The data that support the findings of this study are available from the corresponding author upon reasonable request.
