# Supplementary material for: Are digital health interventions valuable to support patients with cancer and caregivers? An umbrella review of web‐based and app‐based supportive care interventions
Source: Cancer Med. 2023 Nov 8;12(23):21436–51. doi: 10.1002/cam4.6695 (PMC10726780; doi:10.1002/cam4.6695)
Supplement: Supplementary file 1 — Appendix 1. Search strategies Appendix 2. Characteristics of the studies Appendix 3. Excluded full text Appendix 4. Original studies [file CAM4-12-21436-s001.docx]

Supplementary material

Appendix 1. Search strategies

Appendix 2. Characteristics of the studies

Appendix 3. Excluded full text

Appendix 4. Original studies

**Appendix 1. Search strategy**

First running of the search: 04/04/2022; Re-run of the search: 11/28/2022

**Pubmed = 2244 references**

(("systematic review"[Title/Abstract] OR "meta-analys*"[Title/Abstract] OR "scoping review"[Title/Abstract]) AND ("Mobile app*"[Title/Abstract] OR "internet"[Title/Abstract] OR "mhealth"[Title/Abstract] OR "mobile health"[Title/Abstract] OR "health App"[Title/Abstract] OR "health application"[Title/Abstract] OR "digital health intervention*"[Title/Abstract] OR "digital intervention*"[Title/Abstract] OR "ehealth"[Title/Abstract] OR "electronic health"[Title/Abstract] OR "multimedia"[Title/Abstract] OR "web-based"[Title/Abstract] OR "online*"[Title/Abstract] OR "telehealth"[Title/Abstract] OR "telemedicine"[Title/Abstract] OR "telenursing"[Title/Abstract] OR "remote"[Title/Abstract] OR "computer*"[Title/Abstract])) AND ("cancer"[Title/Abstract] OR "neoplasm"[Title/Abstract] OR "leukemia"[Title/Abstract] OR "lymphoma"[Title/Abstract] OR "myeloma"[Title/Abstract] OR "sarcoma"[Title/Abstract]))

Restricted to:

- Publication date: from 2000

- Publication language: English, French, Spanish

Re-run on the 11/28/2022: n = 2469

**Embase = 3020 references**

('systematic review':ti,ab,kw OR 'meta-analys*':ti,ab,kw OR 'scoping review':ti,ab,kw) AND ('mobile app*':ti,ab,kw OR 'internet':ti,ab,kw OR 'mhealth':ti,ab,kw OR 'mobile health':ti,ab,kw OR 'health app':ti,ab,kw OR 'health application':ti,ab,kw OR 'digital health intervention*':ti,ab,kw OR 'digital intervention*':ti,ab,kw OR 'ehealth':ti,ab,kw OR 'electronic health':ti,ab,kw OR 'multimedia':ti,ab,kw OR 'web-based':ti,ab,kw OR 'online*':ti,ab,kw OR 'telehealth':ti,ab,kw OR 'telemedicine':ti,ab,kw OR 'telenursing':ti,ab,kw OR 'remote':ti,ab,kw OR 'computer*':ti,ab,kw) AND ('cancer':ti,ab,kw OR 'neoplasm':ti,ab,kw OR 'leukemia':ti,ab,kw OR 'lymphoma':ti,ab,kw OR 'myeloma':ti,ab,kw OR 'sarcoma':ti,ab,kw)

Restricted to:

- Publication date: from 2000

- Publication language: English, French, Spanish

Re-run on the 11/28/2022: n = 3331

**CINAHL = 1212 references (with 1206 exported)**

(“systematic review” OR “meta-analys*” OR “scoping review”) AND (“Mobile app*” OR “internet” OR “mhealth” OR “mobile health” OR “health App” OR “health application” OR “digital health intervention*” OR “digital intervention*” OR “ehealth” OR “electronic health” OR “multimedia” OR “web-based” OR “online*” OR “telehealth” OR “telemedicine” OR “telenursing” OR “remote” OR “computer*”) AND ("cancer" OR “neoplasm” OR “leukemia” OR “lymphoma” OR “myeloma” OR “sarcoma”)

Restricted to:

- Publication date: from 2000

- Publication language: English, French, Spanish

Re-run on the 11/28/2022: n = 105

**Cochrane Library = 40**

(“systematic review” OR “meta-analys*” OR “scoping review”) AND (“Mobile app*” OR “internet” OR “mhealth” OR “mobile health” OR “health App” OR “health application” OR “digital health intervention*” OR “digital intervention*” OR “ehealth” OR “electronic health” OR “multimedia” OR “web-based” OR “online*” OR “telehealth” OR “telemedicine” OR “telenursing” OR “remote” OR “computer*”) AND ("cancer" OR “neoplasm” OR “leukemia” OR “lymphoma” OR “myeloma” OR “sarcoma”)

Restricted to:

- Keywords in title, abstract, or keywords
- Publication date: from 2000
- No language was specified in research filters

Re-run on the 11/28/2022: n = 43.

**PsycINFO = 310**

(“systematic review” OR “meta-analys*” OR “scoping review”) AND (“Mobile app*” OR “internet” OR “mhealth” OR “mobile health” OR “health App” OR “health application” OR “digital health intervention*” OR “digital intervention*” OR “ehealth” OR “electronic health” OR “multimedia” OR “web-based” OR “online*” OR “telehealth” OR “telemedicine” OR “telenursing” OR “remote” OR “computer*”) AND ("cancer" OR “neoplasm” OR “leukemia” OR “lymphoma” OR “myeloma” OR “sarcoma”)

Restricted to:

- Publication date: from 2000

- Publication language: English, French, Spanish

Re-run on the 11/28/2022: n = 351.

**Google Scholar = 25 (11/28/2022)**

allintitle: ((Mhealth OR ((mobile OR health) AND (app OR application)) OR “digital intervention” OR ehealth OR web-based OR online)) AND (cancer OR neoplasm OR leukemia OR lymphoma OR myeloma OR sarcoma) AND ("systematic review" OR "meta-analysis")

Restricted to:

- Publication date: from 2000 to 2022

**Appendix 2. Characteristics of the Included Studies**

| **Author** | **Country** | **Databases** | **Date range** | **Type of review** | **Number of articles**  **(Number of studies)** | **Population** | **Sample size** | **Source of funding** |
| --- | --- | --- | --- | --- | --- | --- | --- | --- |
| Buneviciene et al. (2021) | Lithuania | Pubmed/MEDLINE, Web of Knowledge | No date range reported. Search realized on July 20, 2019 | Systematic review and meta-analysis | 25 | Adults with different cancer types | 2634 | Research Council of Lithuania, Lithuania, Grant No. P-COV-20-50 |
| Dorri et al. (2019) | Iran | PubMed, Embase, Cochrane Central Register of Controlled Trials, IEEE, Web of Science, Scopus, Science Direct, Google Scholar | No date range reported. Database searching in July 2018 | Systematic review | 16 | Adults with breast cancer | 2304 (1528 without CG) | None |
| Ester et al. (2021) | Canada | MEDLINE and Epub Ahead of Print, In-Process and Other Non-Indexed Citations and Daily, Embase, CENTRAL, CINAHL, SPORTDiscus, Scopus | No date range reported. Database searching on December 18, 2019 (update on January 7, 2021) | Systematic review | 67 (71) | Adults with different cancer types | 6655 | None |
| Goliță et al. (2019) | Romania | PubMed, CINAHL, PsycINFO, Web of Science | Published before March 2019. Database search on April 1, 2019 | Systematic review | 19 (21) | Adults with different cancer types | 4084 | None |
| Haberlin et al. (2018) | Ireland | PubMed, CINAHL, Embase, PsycINFO, Web of Science, Scopus | Until March 2017 | Systematic review | 10 | Adults with different cancer types | 1994 | Studentship grant from Trinity College, Dublin, during the conduct of the study |
| Hong et al. (2021) | South Korea | PUBMED, Embase, Cochrane, CINAHL, Web of Science, PsycArticles, Scopus, PQDT Global | Until September 16, 2019 | Systematic review and meta-analysis | 8 | Childhood cancer survivors | 206 (without CG) | Basic Science Research Program, National Research Foundation of Korea (NRF), and the Ministry of Education |
| Huang et al. (2019) | China | CENTRAL, Embase, MEDLINE, Physiotherapy Evidence Database, Web of Science, China National Knowledge Infrastructure, Wan Fang, Vip database, ClinicalTrials.gov | Until January 2019 | Systematic review and meta-analysis | 13 | Adults with different cancer types | 749 (without CG) | None |
| Kaltenbaugh et al. (2015) | USA | PubMed, EBSCOhost CINAHL, Ovid PsycINFO, Inspec | Until February 1, 2014 | Systematic review | 6 | Caregivers of adult patients with different cancer types | 924 | Grant from the Greater Pittsburgh Nursing Research Conference |
| Kamalumpundi et al. (2022) | USA | PubMed, PsycINFO, CINAHL, Scopus | From 1991 to November 2019 | Systematic review and meta-analysis | 23 (19 for the meta-analysis) | Adults with different advanced cancer types | 2558 | None |
| Kang et al. (2018) | South Korea | CINAHL, Cochrane, EBSCO, PROQUEST, PubMed, PQDT | Until December 2015 | Systematic review | 4 | Adults with different cancer types and their partners (for 3/4 studies) | 195 | Korea Research Foundation grant funded by the Korean Government (MOEHRD, Basic Research Promotion Fund) |
| Kim et al. (2019) | South Korea | CINAHL, Web of Science, PubMed, PsycINFO | Until May 2018 | Systematic review | 7 | Adults with different cancer types | 714 | Not specified |
| Kiss et al. (2019) | Australia | MEDLINE Complete, Scopus, CINAHL, Embase, Cochrane Library, SPORTDiscus | From 1973 to July 2018 | Systematic review | 16 (18) | Adults with different cancer types | 2684 | None |
| McAlpine et al. (2015) | Australia | MEDLINE, PsychINFO, Chochrane Central Register of Controlled Trials, CINAHL,  Inspec, Computers and Applied Science | Until February 2014 | Systematic review | 14 | Adults with different cancer types | 2351 | University of Melbourne’s “Major Research Projects and Initiatives Development and Support” fund |
| Qan'ir et al. (2019) | USA | CINAHL, Embase, “Library and Information Science Abstracts,” “Library, Information Science and Technology Abstracts,” “Library and Information Science Source,” PsychINFO, Pubmed | Between January 1, 2000, and September 15, 2018 | Systematic review | 10 | Adults with breast, colorectal, or prostate cancer | 1124 | R01 NR016990-01A1 and UNC Lineberger Comprehensive Cancer Center University Cancer Research Fund |
| Ramsey et al. (2020) | USA | PubMed, CINAHL, Embase, PsycINFO, IEEEXplore, Cochrane Library | Not reported | Systematic review | 21 | Pediatric patients undergoing active treatment (k = 8) or childhood cancer survivors (k = 13) | 1506 | Agency for Healthcare Research and Quality |
| Seiler et al. (2017) | USA | PubMed, MEDLINE, Embase, Cochrane Library | Up to November 2016 | Systematic review and meta-analysis | 9 (+ 6 study protocols) | Adults with different cancer types | 1580 | Swiss National Science Foundation (SNSF) |
| Singleton et al. (2022) | Australia | PreMEDLINE, MEDLINE, Cochrane Central Registry of Controlled Trials, Embase, PsycINFO, Allied and Complementary Medicine, Scopus, Web of Science, CINAHL | Up to October 19, 2019 | Systematic review and meta-analysis | 32 (36) | Adults with breast cancer | 4790 | None |
| Wan et al. (2022) | Singapore | PubMed, PsycINFO, Embase, Scopus, CINAHL, CNKI | Up to December 8, 2021 | Systematic review and meta-analysis | 19 | Adults with different cancer types (mostly colorectal cancer) | 1386 | Singapore Cancer Society Research Grant 2018 |
| Zhang et al. (2022) | China | Web of Science, PubMed, Embase, Cochrane Library | Up to 2021 | Systematic review and meta-analysis | 9 | Adults with different cancer types | 2471 | None |
| Zheng et al. (2020) | China | MEDLINE, Embase, Cochrane, CINAHL, Scopus, PsycINFO | Up to August 2019 | Systematic review and meta-analysis | 13 | Adults with different cancer types | 487 | Joint Funds for the Innovation of Science and Technology, Fujian Province, and Key Project for Youth Academic Talents from the Health and Family Planning Commission of Fujian Province |

CG: Control group

**Appendix 2. Characteristics of the Included Studies**

| **Author** | **Phenomena of interest** | **Interventions** | **RoB assessment** | **Outcome measured** |
| --- | --- | --- | --- | --- |
| Buneviciene et al. (2021) | HRQoL | PA or fitness (n = 9), cognitive behavioral therapy or behavioral change (n = 6 ), mindfulness or stress management (n = 3), social support (n = 2), information/psychoeducation (n = 2), weight management (n = 2), pain management (n = 1)  Type of interventions Web-based (n = 6)  Monitoring device (n = 6) App (n = 9) Web and mobile based (n =1) Virtualized CBT (n =1) Videoconference (n =1) Unknown (n =1) | No overall rating reported.   Selection bias (random sequence): 15/25 low; 2/12 unclear; 8 high Selection bias (allocation concealment): 3/25 low, 13/25 unclear; 9/25 high Blinding of participants: 8/25 low, 5/25 unclear, 12/25 high Incomplete outcome data : 25/25 low  Selective outcome reporting : 25/25 low Other sources of bias: 25/25 low | HRQoL |
| Dorri et al. (2019) | PA | PA (9 web-based, 5 mobile-based, 2 web and mobile-based) | RCT: low (n = 8) and unclear (n = 2) Non-RCT: low (n = 3) and high (n = 2) | PA (change in the level of PA or physical functions, time lapsed during PA, compliance with PA recommendations, consumed energy) |
| Ester et al. (2021) | PA and the theory used to design the intervention | PA with 1 to 5 technology components (2 components being the most common: 40%). Technology components used were wearable devices (41/67, 61%), websites (32/67, 48%), text messages (19/67, 28%), mobile apps (18/67, 27%), and email (15/67, 22%) | RCT: low (n = 4) and unclear (n = 41). Non-RCT (n = 26): no overall rating reported. | PA (change in the level of PA) |
| Goliță et al. (2019) | Emotional distress and HRQoL | Web-based interventions focusing on stress management (n = 6), emotional well-being or psychological distress (n = 6), fatigue (n = 2), sexual functioning (n = 1), fear of cancer recurrence (n = 1), decreasing the impact of treatment induced menopausal symptoms (n = 1) or with several components (n = 2). | Strong (n = 6), moderate (n = 8) and weak (n = 5) | Emotional distress and HRQoL |
| Haberlin et al. (2018) | PA | PA web-based (n = 5), web and mobile-based (n = 4) and e-mail-based (n = 1). | RCTs: low (n = 4), unclear RoB (n = 3) non-RCTs: poor quality (n = 3) | PA self-report, objective or direct methods, PA expressed in a number of ways |
| Hong et al. (2021) | HRQoL and MVPA | Web-based (n = 6), mobile and social network based (n = 1), and social network based (n = 1) | RCT: low (n = 3) Non-RCT: unclear (n = 2), high (n = 2) | HRQoL and MVPA |
| Huang et al. (2019) | Fatigue, depression, anxiety, sleep quality, life quality | Web-based knowledge-related disease, psychological adjustment, and life management (n = 13) | No overall rating reported. Studies had low to moderate risk (n = 13) regarding the criteria assessed. | Fatigue |
| Kaltenbaugh et al. (2015) | Physical, social, psychological, and financial quality of life | Web based multi-component interventions (n = 6) | Weak (n = 6) | Psychological (positive affect, mood, burden, coping strategies, stress) |
| Kamalumpundi et al. (2022) | Emotional management | Web-based (n = 4) and mobile-based (n = 19) interventions targeting emotional management | 6 non-RCT: 1 high RoB, 5 moderate RoB 17 RCT: 14 high RoB, remaining with potential bias | Psychological (depression, anxiety, distress, despair, negative mood, stress) |
| Kang et al. (2018) | Sexual health | Web-based multi-component interventions (education, interactive methods, cognitive behavior therapy, tailored information, and self-monitoring interventions) | All studies fulfilled the quality assessment (except for 3 that did not justify their sample size). | Sexual function (physical outcomes, psychological or sexual distress) |
| Kim et al. (2019) | Cognitive function | Web-based (n = 4) or computerized (n = 3) interventions targeting memory (n = 5), attention (n = 4), and executive function (n = 1). | Not realized | Cognitive function (eg, memory, attention, brain speed, recognition skills) |
| Kiss et al. (2019) | Nutrition and PA | Web-based (n = 9), mobile-based (n = 3), wearable devices (n = 3), and digital video disk (n = 1) interventions on PA and nutrition. | No overall rating reported | Dietary behaviors and PA (several measures) |
| McAlpine et al. (2015) | HRQoL and directly related outcomes (mood, symptom burden, social support) | Web-based (n = 13), telephone-based symptom monitoring (n = 1) | Not realized | Mood disturbance (depression, anxiety, PTSD), HRQoL, and cancer-related symptoms |
| Qan'ir et al. (2019) | Anxiety, depression, HRQoL | Self‐guided websites (n = 5), web‐based programs supported by medical professionals (n = 4), and mobile health app with clinical nurse supervision (n = 1) | High risk (n = 10) | Anxiety, depression, HRQoL |
| Ramsey et al. (2020) | Emotional distress, health behaviors, health-related outcomes, neurocognitive functioning, perception, attitudes, and concerns toward digital health interventions | eHealth (n = 16): social robotic-assisted therapy (n =1) cognitive rehabilitation (n = 4), web-based resources (n = 5), video games to improve treatment adherence (n = 4), and virtual reality (n = 2) mHealth (n = 5): wearable technology (n = 2), combination of wearable technology and social media (n = 1), combination of web-and-text messaging (n = 1), and gamified electronic monitoring smartphone app (n = 1) | RCT (n = 13): low (n = 3), moderate (n = 3), unclear (n = 7) Observational studies: 3/9 (n = 2), 4/9 (n = 2), 5/9 (n = 1), 6/9 (n = 2), 7/9 (n = 1) | Emotional distress (anger, anxiety, depression, perceived stress, posttraumatic stress), health behaviors (PA, activities of daily living, weight management, smoking cessation, alcohol use), health-related outcomes (fatigue, pain, and markers of physical functioning), cancer knowledge, self-efficacy, HRQoL, neurocognitive functioning, perception, attitudes and concerns toward digital health interventions |
| Seiler et al. (2017) | Cancer related fatigue | Educational programs (n =1) and behavior change interventions, including psycho‐educational modules on fatigue, anxiety, depression, diet, exercise, sleep, and social relationships (n = 4) Mindfulness‐based cognitive therapy (n = 1) and an imagery‐based behavioral intervention (n = 1). Two studies investigated a web‐based exercise intervention (n = 2) | Unclear (n = 5), Low (n = 4) | Cancer-related fatigue |
| Singleton et al. (2022) | HRQoL, self-efficacy, and mental (anxiety, depression, and distress) or physical (PA, nutrition, and fatigue) quality of life | All interventions were multicomponent and promoted self-management. Patient-directed eHealth interventions (including but not limited to e-mail, videoconferencing, videos, activity trackers, website, podcast, chatroom, mobile applications, or text messages).  24/32 used a web-based intervention, 6/32 a mobile-app, 1 used both, and 1 used videocall | Low (n = 3), unclear (n = 19), high (n = 10) (according to supplementary data) | HRQoL, self-efficacy, and mental (anxiety, depression, and distress) or physical (PA, nutrition, and fatigue) quality of life |
| Wan et al. (2022) | Psychosocial outcomes (self-efficacy, anxiety, depression, distress, quality of life), cancer-specific clinical outcomes (treatment side-effects or symptoms) | Web-based interventions with various psychosocial interventions including psychoeducation, acceptance and commitment therapy, mindfulness, cognitive behavioral therapy, peer support, counselling and stress management. | No overall rating reported.   For RCTs Selection bias (random sequence): 10/12 low, 2/12 unclear Selection bias (allocation concealment): 9/12 low, 2/12 unclear, 1/12 high Performance bias: 3 low, 5 unclear, 4 high Detection bias: 8 low, 1 unclear, 3 high Attrition bias: 12/12 low Reporting bias: 10 low, 2 unclear  For non RCTs Confounding bias: 3 low, 3 moderate, 1 high Selection bias: 5 low, 2 high Classification of intervention bias: 6 low, 1 high Deviations from interventions: 4 low, 2 moderate, 1 missing information Missing data bias: 6 low, 1 high Bias in measurement: 1 low, 4 moderate, 2 high Bias in reporting results: 7 low | Anxiety, depression, psychological distress, cancer-specific distress, self-efficacy, quality of life |
| Zhang et al. (2022) | Cancer distress, quality of life, depression, self-efficacy | Multi-component web-based interventions (n = 8) and mobiles app (n = 1) | No overall rating reported.  9/9 studies presented a low risk of selection bias, reporting bias and other bias 9/9 studies presented a high risk of performance bias 7/9 studies presented a unclear risk of detection bias and 2/9 a low risk 6/9 studies presented a low risk of attrition bias and 3/9 a high risk | Cancer distress, post-traumatic stress, HRQoL, depression, self-efficacy |
| Zheng et al. (2020) | Cancer pain | 11 mobile app with multiple components and 2 Skype-based interventions. The contents of the interventions were not described in detail | Low (n = 13) | Pain |

RoB: Risk of bias, HRQoL: Health Related Quality of Life, PA: physical activity, MVPA: moderate-to-vigorous physical activity, RCT: randomized controlled trial.

**Appendix 3. Excluded full texts**

| **N°** | **Full-text reference** | **Reason for exclusion** |
| --- | --- | --- |
| 1 | Agboola, S. O., Ju, W., Elfiky, A., Kvedar, J. C., & Jethwani, K. (2015). The effect of technology-based interventions on pain, depression, and quality of life in patients with cancer: a systematic review of randomized controlled trials. Journal of medical Internet research, 17(3), e4009. | Telehealth |
| 2 | Akingbade, O., Nguyen, K. T., & Chow, K. M. (2022). Effect of mHealth interventions on psychological issues experienced by women undergoing chemotherapy for breast cancer: A systematic review and meta‐analysis. Journal of Clinical Nursing. | Interventions are not only digital |
| 3 | Akingbade, O., Nguyen, K. T., & Chow, K. M. (2022). Effect of mHealth interventions on psychological issues experienced by women diagnosed with breast cancer receiving chemotherapy: A systematic review and meta-analysis. medRxiv. | Interventions are not only digital |
| 4 | Ayyoubzadeh, S. M., R Niakan Kalhori, S., Shirkhoda, M., Mohammadzadeh, N., & Esmaeili, M. (2020). Supporting colorectal cancer survivors using eHealth: a systematic review and framework suggestion. *Supportive Care in Cancer*, *28*(8), 3543-3555. | Not only supportive care |
| 5 | Binarelli, G., Joly, F., Tron, L., Lefevre Arbogast, S., & Lange, M. (2021). Management of Cancer-Related Cognitive Impairment: A Systematic Review of Computerized Cognitive Stimulation and Computerized Physical Activity. *Cancers*, *13*(20), 5161. | Interventions are not only digital |
| 6 | Chandeying, N., & Thongseiratch, T. (2021). Online Interventions to Improve Mental Health of Pediatric, Adolescent, and Young Adult Cancer Survivors: A Systematic Review and Meta-Analysis. *Frontiers in psychiatry*, *12*. | Interventions are not only digital |
| 7 | Cheng, L., Duan, M., Mao, X., Ge, Y., Wang, Y., & Huang, H. (2021). The effect of digital health technologies on managing symptoms across pediatric cancer continuum: A systematic review. *International journal of nursing sciences*, *8*(1), 22-29. | Not only supportive care |
| 8 | Cheng, L., Liu, F., Mao, X., Peng, W., Wang, Y., Huang, H., ... & Yuan, C. (2022). The Pediatric Cancer Survivors' User Experiences With Digital Health Interventions: A Systematic Review of Qualitative Data. *Cancer Nursing*, *45*(1), E68-E82. | Not only supportive care |
| 9 | Christopherson, U., Wells, S., Parker, N., Lyons, E., Swartz, M., Blozinski, A., ... & Swartz, M. (2020). The use of Videoconferencing and Active Video Games to Improve Physical Function and Health Outcomes Among Adolescent and Young Adult Cancer Survivors: A Systematic Review. *Archives of Physical Medicine and Rehabilitation*, *101*(12), e159. | Congress abstract |
| 10 | Cruz, F. O. A. M., Vilela, R. A., Ferreira, E. B., Melo, N. S., & Dos Reis, P. E. D. (2019). Evidence on the use of mobile apps during the treatment of breast cancer: systematic review. *JMIR mHealth and uHealth*, *7*(8), e13245. | Not only supportive care |
| 11 | Curry, J., Patterson, M., Greenley, S., Pearson, M., & Forbes, C. C. (2021). Feasibility, acceptability, and efficacy of online supportive care for individuals living with and beyond lung cancer: A systematic review. *Supportive Care in Cancer*, *29*(11), 6995-7011. | Not only supportive care |
| 12 | Darley, A., Coughlan, B., & Furlong, E. (2021). People with cancer and their family caregivers’ personal experience of using supportive eHealth technology: a narrative review. *European Journal of Oncology Nursing*, *54*, 102030. | Reported studies are not only interventional research |
| 13 | Delemere, E., & Maguire, R. (2021). The role of Connected Health technologies in supporting families affected by paediatric cancer: A systematic review. *Psycho‐Oncology*, *30*(1), 3-15. | Not only supportive care |
| 14 | Escriva Boulley, G., Leroy, T., Bernetière, C., Paquienseguy, F., Desfriches‐Doria, O., & Préau, M. (2018). Digital health interventions to help living with cancer: a systematic review of participants' engagement and psychosocial effects. *Psycho‐oncology*, *27*(12), 2677-2686. | Interventions are not only digital |
| 15 | Forbes, C. C., Finlay, A., McIntosh, M., Siddiquee, S., & Short, C. E. (2019). A systematic review of the feasibility, acceptability, and efficacy of online supportive care interventions targeting men with a history of prostate cancer. *Journal of Cancer Survivorship*, *13*(1), 75-96. | Not only supportive care |
| 16 | Fung, J. Y. T., Lim, H., Vongsirimas, N., & Klainin-Yobas, P. (2022). Effectiveness of eHealth mindfulness-based interventions on cancer-related symptoms among cancer patients and survivors: A systematic review and meta-analysis. *Journal of Telemedicine and Telecare*, 1357633X221078490. | Article not available |
| 17 | Furness, K., Huggins, C., Sarkies, M., Croagh, D., & Haines, T. (2020). Does the method of delivering e-health behaviour change interventions in patients with/or survivors of cancer impact engagement, health behaviours and health outcomes? a systematic review and meta-analysis. *Clinical Nutrition ESPEN*, *40*, 559. | Interventions are not only digital |
| 18 | Furness, K., Sarkies, M. N., Huggins, C. E., Croagh, D., & Haines, T. P. (2020). Impact of the method of delivering electronic health behavior change interventions in survivors of cancer on engagement, health behaviors, and health outcomes: systematic review and meta-analysis. *Journal of medical Internet research*, *22*(6), e16112. | Congress abstract |
| 19 | Gitonga, I., Desmond, D., Duda, N., & Maguire, R. (2022). Impact of connected health interventions on psychological wellbeing and quality of life in patients with cancer: A systematic review and meta‐analysis. *Psycho‐Oncology*, *31*(10), 1621-1636. | Interventions are not only digital |
| 20 | Goode, A. D., Lawler, S. P., Brakenridge, C. L., Reeves, M. M., & Eakin, E. G. (2015). Telephone, print, and Web-based interventions for physical activity, diet, and weight control among cancer survivors: a systematic review. *Journal of Cancer Survivorship*, *9*(4), 660-682. | Interventions are not only digital |
| 21 | Gysels, M., & Higginson, I. J. (2007). Interactive technologies and videotapes for patient education in cancer care: systematic review and meta-analysis of randomised trials. *Supportive care in cancer*, *15*(1), 7-20. | Not only supportive care |
| 22 | Hasnan, S., Aggarwal, S., Mohammadi, L., & Koczwara, B. (2022). Barriers and enablers of uptake and adherence to digital health interventions in older patients with cancer: A systematic review. *Journal of Geriatric Oncology*. | Qualitative studies |
| 23 | Hernandez Silva, E., Lawler, S., & Langbecker, D. (2019). The effectiveness of mHealth for self-management in improving pain, psychological distress, fatigue, and sleep in cancer survivors: a systematic review. *Journal of Cancer Survivorship*, *13*(1), 97-107. | Telehealth |
| 24 | Heynsbergh, N., Heckel, L., Botti, M., & Livingston, P. M. (2018). Feasibility, useability and acceptability of technology-based interventions for informal cancer carers: a systematic review. *BMC cancer*, *18*(1), 1-11. | Not only supportive care |
| 25 | Hong, Y. A., Hossain, M. M., & Chou, W. Y. S. (2020). Digital interventions to facilitate patient‐provider communication in cancer care: A systematic review. *Psycho‐Oncology*, *29*(4), 591-603. | Not only supportive care |
| 26 | Hopstaken, J. S., Verweij, L., van Laarhoven, C. J., Blijlevens, N. M., Stommel, M. W., & Hermens, R. P. (2021). Effect of digital care platforms on quality of care for oncological patients and barriers and facilitators for their implementation: Systematic review. *Journal of Medical Internet Research*, *23*(9), e28869. | Not only supportive care |
| 27 | Huang, Y., Li, Q., Zhou, F., & Song, J. (2022). Effectiveness of internet-based support interventions on patients with breast cancer: a systematic review and narrative synthesis. *BMJ open*, *12*(5), e057664. | Interventions are not only digital |
| 28 | Ibeggazene, S., Turner, R., Rosario, D., & Bourke, L. (2021). Remote interventions to improve exercise behaviour in sedentary people living with and beyond cancer: a systematic review and meta-analysis. *BMC cancer*, *21*(1), 1-10. | Interventions are not only digital |
| 29 | Ihrig, A., Karschuck, P., Haun, M. W., Thomas, C., & Huber, J. (2020). Online peer-to-peer support for persons affected by prostate cancer: a systematic review. *Patient Education and Counseling*, *103*(10), 2107-2115. | Reported studies are not only interventional research |
| 30 | Kane, K., Kennedy, F., Absolom, K., Harley, C., & Velikova, G. (2019). Living better with advanced breast cancer (LIBERATE)-Exploring the effect of online and technology-assisted supportive interventions on the quality of life of patients with advanced cancer: A systematic review. *Psycho-Oncology.* 28: 15-16. | Congress abstract |
| 31 | Kane, K., Kennedy, F., Absolom, K. L., Harley, C., & Velikova, G. (2021). Quality of life support in advanced cancer—web and technological interventions: systematic review and narrative synthesis. *BMJ Supportive & Palliative Care*. | Not only supportive care |
| 32 | Keikha, L., Maserat, E., & Mohammadzadeh, Z. (2022). Telerehabilitation and monitoring physical activity in patient with breast cancer: Systematic review. *Iranian Journal of Nursing and Midwifery Research*, *27*(1), 8. | Interventions are not only digital |
| 33 | Kelley, M. M., Kue, J., Brophy, L., Peabody, A. L., Foraker, R. E., Yen, P. Y., & Tucker, S. (2021). Mobile Health Applications, Cancer Survivors and Lifestyle Modification: An Integrative Review. *Computers, informatics, nursing: CIN*, *39*(11), 755. | Not a systematic review of scientific studies |
| 34 | Khoo, S., Mohbin, N., Ansari, P., Al-Kitani, M., & Müller, A. M. (2021). mHealth interventions to address physical activity and sedentary behavior in cancer survivors: a systematic review. *International journal of environmental research and public health*, *18*(11), 5798. | Reported studies are not only interventional research |
| 35 | Kim, A. R., & Park, H. A. (2015). Web-based Self-management Support Interventions for Cancer Survivors: A Systematic Review and Meta-analyses. *MedInfo*, 142-147. | Not only supportive care |
| 36 | Kopp, L. M., Gastelum, Z., Guerrero, C. H., Howe, C. L., Hingorani, P., & Hingle, M. (2017). Lifestyle behavior interventions delivered using technology in childhood, adolescent, and young adult cancer survivors: a systematic review. *Pediatric blood & cancer*, *64*(1), 13-17. | Interventions are not only digital |
| 37 | Li, J., Zhu, C., Liu, C., Su, Y., Peng, X., & Hu, X. (2022). Effectiveness of eHealth interventions for cancer‐related pain, fatigue, and sleep disorders in cancer survivors: A systematic review and meta‐analysis of randomized controlled trials. *Journal of Nursing Scholarship*, *54*(2), 184-190. | Interventions are not only digital |
| 38 | Li, J., Zhu, C., Liu, C., Su, Y., Peng, X., & Hu, X. (2022). Effectiveness of eHealth interventions for cancer‐related pain, fatigue, and sleep disorders in cancer survivors: A systematic review and meta‐analysis of randomized controlled trials. *Journal of Nursing Scholarship*, *54*(2), 184-190. | Interventions are not only digital |
| 39 | Li, Y., Li, J., Zhang, Y., Ding, Y., & Hu, X. (2022). The effectiveness of e-Health interventions on caregiver burden, depression, and quality of life in informal caregivers of patients with cancer: A systematic review and meta-analysis of randomized controlled trials. *International journal of nursing studies*, 104179. | Interventions are not only digital |
| 40 | Lim, N. L., & Shorey, S. (2019). Effectiveness of technology‐based educational interventions on the empowerment related outcomes of children and young adults with cancer: A quantitative systematic review. *Journal of Advanced Nursing*, *75*(10), 2072-2084. | Not only supportive care |
| 41 | Lin, H., Ye, M., Chan, S. W. C., Zhu, J., & He, H. (2020). The effectiveness of online interventions for patients with gynecological cancer: an integrative review. *Gynecologic Oncology*, *158*(1), 143-152. | Not only supportive care |
| 42 | Liu, T., Xu, J., Cheng, H., Zhang, Y., Wang, S., Lin, L., & Tian, L. (2022). Effects of internet-based cognitive behavioral therapy on anxiety and depression symptoms in cancer patients: A meta-analysis. *General Hospital Psychiatry*. | Interventions are not only digital |
| 43 | Lopez-Rodriguez, M. M., Fernández-Millan, A., Ruiz-Fernández, M. D., Dobarrio-Sanz, I., & Fernández-Medina, I. M. (2020). New technologies to improve pain, anxiety and depression in children and adolescents with cancer: a systematic review. *International journal of environmental research and public health*, *17*(10), 3563. | Not only supportive care |
| 44 | Luo, X., Chen, Y., Chen, J., Zhang, Y., Li, M., Xiong, C., & Yan, J. (2021). Effectiveness of mobile health-based self-management interventions in breast cancer patients: a meta-analysis. *Supportive Care in Cancer*, 1-24. | Not only supportive care |
| 45 | Marthick, M., McGregor, D., Alison, J., Cheema, B., Dhillon, H., & Shaw, T. (2021). Supportive care interventions for people with cancer assisted by digital technology: systematic review. *Journal of medical Internet research*, *23*(10), e24722. | Not a systematic review or a meta-analysis |
| 46 | Matis, J., Svetlak, M., Slezackova, A., Svoboda, M., & Šumec, R. (2020). Mindfulness-based programs for patients with cancer via eHealth and mobile health: systematic review and synthesis of quantitative research. *Journal of medical Internet research*, *22*(11), e20709. | Interventions are not only digital |
| 47 | McCann, L., McMillan, K. A., & Pugh, G. (2019). Digital interventions to support adolescents and young adults with cancer: systematic review. *JMIR cancer*, *5*(2), e12071. | Not only supportive care |
| 48 | McCracken, A., Harrison, J., & Hill, J. (2021). Self-guided technology to improve health-related behaviour and quality of life in people with cancer. *British Journal of Community Nursing*, *26*(9), 434-437. | Not a systematic review or a meta-analysis |
| 49 | Moradian, S., Voelker, N., Brown, C., Liu, G., & Howell, D. (2018). Effectiveness of Internet-based interventions in managing chemotherapy-related symptoms in patients with cancer: a systematic literature review. *Supportive Care in Cancer*, *26*(2), 361-374. | Not only supportive care |
| 50 | Ozturk, C. S., & Toruner, E. K. (2022). Effectiveness of technology‐based psychosocial interventions for child, adolescents and young adults undergoing cancer treatment: A meta‐analysis of randomised controlled trials. *European Journal of Cancer Care*, *31*(1), e13515. | Interventions are not only digital |
| 51 | Pfirrmann, D., & Simon, P. (2016). Internet as an appropriate medium for training support in cancer patients-a systematic review of the literature. *Oncology research, 39:* 153-153*.* | Congress abstract |
| 52 | Post, K. E., & Flanagan, J. (2016). Web based survivorship interventions for women with breast cancer: an integrative review. *European Journal of Oncology Nursing*, *25*, 90-99. | Participants are not only cancer patients |
| 53 | Richards, R., Kinnersley, P., Brain, K., McCutchan, G., Staffurth, J., & Wood, F. (2018). Use of mobile devices to help cancer patients meet their information needs in non-inpatient settings: systematic review. *JMIR mHealth and uHealth*, *6*(12), e10026. | Not only supportive care |
| 54 | Roberts, A. L., Fisher, A., Smith, L., Heinrich, M., & Potts, H. W. (2017). Digital health behaviour change interventions targeting physical activity and diet in cancer survivors: a systematic review and meta-analysis. *Journal of Cancer Survivorship*, *11*(6), 704-719. | Interventions are not only digital |
| 55 | Saeidzadeh, S., Kamalumpundi, V., Chi, N. C., Nair, R., & Gilbertson-White, S. (2021). Web and mobile-based symptom management interventions for physical symptoms of people with advanced cancer: A systematic review and meta-analysis. *Palliative Medicine*, *35*(6), 1020-1038. | Not only supportive care |
| 56 | Salonen, A., Ryhänen, A. M., & Leino-Kilpi, H. (2014). Educational benefits of Internet and computer-based programmes for prostate cancer patients: a systematic review. *Patient Education and Counseling*, *94*(1), 10-19. | Not only supportive care |
| 57 | Shaffer, K. M., Tigershtrom, A., Badr, H., Benvengo, S., Hernandez, M., & Ritterband, L. M. (2020). Dyadic psychosocial eHealth interventions: systematic scoping review. *Journal of medical Internet research*, *22*(3), e15509. | Participants are not only cancer patients |
| 58 | Suh, S. R. (2017). Effects of nurse-led telephone-based supportive interventions for patients with cancer: A meta-analysis. *Number 4/July 2017*, *44*(4), E168-E184. | Interventions are not only digital |
| 59 | Sotirova, M. B., McCaughan, E. M., Ramsey, L., Flannagan, C., Kerr, D. P., O’Connor, S. R., ... & Wilson, I. M. (2021). Acceptability of online exercise-based interventions after breast cancer surgery: systematic review and narrative synthesis. *Journal of Cancer Survivorship*, *15*(2), 281-310. | Not only supportive care |
| 60 | Triberti, S., Savioni, L., Sebri, V., & Pravettoni, G. (2019). eHealth for improving quality of life in breast cancer patients: a systematic review. *Cancer treatment reviews*, *74*, 1-14. | Reported studies are not only interventional research |
| 61 | Ugalde, A., Haynes, K., Boltong, A., White, V., Krishnasamy, M., Schofield, P., ... & Livingston, P. (2017). Self-guided interventions for managing psychological distress in people with cancer–a systematic review. *Patient education and counseling*, *100*(5), 846-857. | Interventions are not only digital |
| 62 | Ugalde, A., Haynes, K., White, V., Krishnasamy, M., Boltong, A., Schofield, P., ... & Livingston, P. (2015, January). Self-guided psychological interventions for people with cancer: a systematic review. In *COSA 2015: Proceedings of the Annual Scientific Meeting. Rare Cancers: Common Goals, 17–19 November 2015, The Federation Conference and Exhibition Centre Hotel Grand Chancellor Hobart* (pp. 1-1). Wiley. | Congress abstrat |
| 63 | van Deursen, L., Versluis, A., van der Vaart, R., Standaar, L., Struijs, J., Chavannes, N., & Aardoom, J. J. (2022). eHealth Interventions for Dutch Cancer Care: Systematic Review Using the Triple Aim Lens. *JMIR cancer*, *8*(2), e37093. | Interventions are not only digital |
| 64 | Ventura, F., Öhlén, J., & Koinberg, I. (2013). An integrative review of supportive e-health programs in cancer care. *European Journal of Oncology Nursing*, *17*(4), 498-507. | Not a systematic review or a meta-analysis |
| 65 | Vergani, L., Marton, G., Pizzoli, S. F. M., Monzani, D., Mazzocco, K., & Pravettoni, G. (2019). Training cognitive functions using mobile apps in breast cancer patients: systematic review. *JMIR mHealth and uHealth*, *7*(3), e10855. | Participants are not only cancer patients |
| 66 | Wang, Y., Lin, Y., Chen, J., Wang, C., Hu, R., & Wu, Y. (2020). Effects of Internet-based psycho-educational interventions on mental health and quality of life among cancer patients: a systematic review and meta-analysis. *Supportive Care in Cancer*, *28*(6), 2541-2552. | Interventions are not only digital |
| 67 | Wijeratne, D. T., Bowman, M., Sharpe, I., Srivastava, S., Jalink, M., & Gyawali, B. (2021). Text messaging in cancer-supportive care: a systematic review. *Cancers*, *13*(14), 3542. | Not only supportive care |
| 68 | Wittenberg-Lyles, E., Oliver, D. P., Demiris, G., Swarz, J., & Rendo, M. (2014). YouTube as a tool for pain management with informal caregivers of cancer patients: a systematic review. *Journal of pain and symptom management*, *48*(6), 1200-1210. | Not a systematic review of scientific studies |
| 69 | Xu, A., Wang, Y., & Wu, X. (2019). Effectiveness of e‐health based self‐management to improve cancer‐related fatigue, self‐efficacy and quality of life in cancer patients: Systematic review and meta‐analysis. *Journal of Advanced Nursing*, *75*(12), 3434-3447. | Interventions are not only digital |
| 70 | Yi, J., & Crawford, B. (2020). PCN95 A Systematic Literature Review of the Use of Mhealth Apps to Improve Quality of Life (QOL) in Cancer Patients. *Value in Health Regional Issues*, *22*, S23. | Congress abstract |
| 71 | Zhang, A., Zebrack, B., Acquati, C., Roth, M., Levin, N. J., Wang, K., & Schwartz, S. (2022). Technology-assisted psychosocial interventions for childhood, adolescent, and young adult cancer survivors: a systematic review and meta-analysis. *Journal of adolescent and young adult oncology*, *11*(1), 6-16. | Interventions are not only digital |
| 72 | Zhang, Q., Zhang, L., Yin, R., Fu, T., Chen, H., & Shen, B. (2018). Effectiveness of telephone‐based interventions on health‐related quality of life and prognostic outcomes in breast cancer patients and survivors—A meta‐analysis. *European journal of cancer care*, *27*(1), e12632. | Interventions are not only digital |
| 73 | Zhu, J., Ebert, L., & Chan, S. W. C. (2017, March). Integrative Review on the Effectiveness of Internet-Based Interactive Programs for Women With Breast Cancer Undergoing Treatment. In *Oncology nursing forum* (Vol. 44, No. 2). | Qualitative studies |

**Appendix 4. Original studies**

| **N°** | **Authors** | **Original study first authors** |
| --- | --- | --- |
| 1 | Seiler et al. (2017) | Abrahams, H. J. G., Gielissen, M. F. M., Goedendorp, M. M., Berends, T., Peters, M. E. W. J., Poort, H., ... & Knoop, H. (2015) |
| 2 | Golita et al. (2019) | Abrahams, H. J., Gielissen, M. F., Donders, R. R., Goedendorp, M. M., van der Wouw, A. J., Verhagen, C. A., & Knoop, H. (2017) |
| 3 | Singleton et al. (2022) | Abrahams, H. J., Gielissen, M. F., Donders, R. R., Goedendorp, M. M., van der Wouw, A. J., Verhagen, C. A., & Knoop, H. (2017) |
| 4 | Buneviciene et al. (2021) | Admiraal, J. M., van der Velden, A. W., Geerling, J. I., Burgerhof, J. G., Bouma, G., Walenkamp, A. M., ... & Reyners, A. K. (2017) |
| 5 | Singleton et al. (2022) | Admiraal, J. M., van der Velden, A. W., Geerling, J. I., Burgerhof, J. G., Bouma, G., Walenkamp, A. M., ... & Reyners, A. K. (2017) |
| 6 | Kamalumpundi et al. (2022) | Alberts, N. M., Hadjistavropoulos, H. D., Dear, B. F., & Titov, N. (2017) |
| 7 | Ramsey et al. (2020) | Alemi, M., Ghanbarzadeh, A., Meghdari, A., & Moghadam, L. J. (2016) |
| 8 | Ester et al. (2021) | Alibhai, S. M., Santa Mina, D., Ritvo, P., Tomlinson, G., Sabiston, C., Krahn, M., ... & Culos-Reed, N. (2019) |
| 9 | Ester et al. (2021) | Allicock, M., Kendzor, D., Sedory, A., Gabriel, K. P., Swartz, M. D., Thomas, P., ... & Rivers, A. (2021) |
| 10 | Singleton et al. (2022) | Atema, V., Van Leeuwen, M., Kieffer, J. M., Oldenburg, H. S., Van Beurden, M., Gerritsma, M. A., ... & Aaronson, N. K. (2019) |
| 11 | Golita et al. (2019) | Atema, V., van Leeuwen, M., Oldenburg, H. S., van Beurden, M., Hunter, M. S., & Aaronson, N. K. (2017) |
| 12 | Wan et al. (2022) | Avci, I. A., Altay, B., Cavusoglu, F., Cal, A., Mumcu, N., Eren, D. C., ... & Buberci, A. (2020) |
| 13 | Ester et al. (2021) | Bade, B. C., Hyer, J. M., Bevill, B. T., Pastis, A., Rojewski, A. M., Toll, B. A., & Silvestri, G. A. (2018) |
| 14 | Zheng et al. (2020) | Bae, W. K., Kwon, J., Lee, H. W., Lee, S. C., Song, E. K., Shim, H., ... & Han, H. S. (2018) |
| 15 | Golita et al. (2019) | Baker, T. B., Hawkins, R., Pingree, S., Roberts, L. J., McDowell, H. E., Shaw, B. R., ... & Gustafson, D. H. (2011) |
| 16 | Singleton et al. (2022) | Baker, T. B., Hawkins, R., Pingree, S., Roberts, L. J., McDowell, H. E., Shaw, B. R., ... & Gustafson, D. H. (2011) |
| 17 | Dorri et al. (2019) | Bantum, E. O. C., Albright, C. L., White, K. K., Berenberg, J. L., Layi, G., Ritter, P. L., ... & Lorig, K. (2014) |
| 18 | Ester et al. (2021) | Bantum, E. O. C., Albright, C. L., White, K. K., Berenberg, J. L., Layi, G., Ritter, P. L., ... & Lorig, K. (2014) |
| 19 | Haberlin et al. (2018) | Bantum, E. O. C., Albright, C. L., White, K. K., Berenberg, J. L., Layi, G., Ritter, P. L., ... & Lorig, K. (2014) |
| 20 | Kiss et al. (2019) | Bantum, E. O. C., Albright, C. L., White, K. K., Berenberg, J. L., Layi, G., Ritter, P. L., ... & Lorig, K. (2014) |
| 21 | Seiler et al. (2017) | Bantum, E. O. C., Albright, C. L., White, K. K., Berenberg, J. L., Layi, G., Ritter, P. L., ... & Lorig, K. (2014) |
| 22 | Golita et al. (2019) | Beatty, L., Kemp, E., Coll, J. R., Turner, J., Butow, P., Milne, D., ... & Koczwara, B. (2019) |
| 23 | Zhang et al. (2022) | Beatty, L., Kemp, E., Coll, J. R., Turner, J., Butow, P., Milne, D., ... & Koczwara, B. (2019) |
| 24 | Wan et al. (2022) | Beatty, L., Kemp, E., Coll, J. R., Turner, J., Butow, P., Milne, D., ... & Koczwara, B. (2019) |
| 25 | Kim et al. (2019) | Becker H, Henneghan AM, Volker DL, Mikan SQ. (2017) |
| 26 | Ester et al. (2021) | Befort, C. A., Klemp, J. R., Austin, H. L., Perri, M. G., Schmitz, K. H., Sullivan, D. K., & Fabian, C. J. (2012) |
| 27 | Hong et al. (2021) | Berg, C. J., Stratton, E., Giblin, J., Esiashvili, N., & Mertens, A. (2014) |
| 28 | Ramsey et al. (2020) | Berg, C. J., Stratton, E., Giblin, J., Esiashvili, N., & Mertens, A. (2014) |
| 29 | Zhang et al. (2022) | Berry, D. L., Hong, F., Halpenny, B., Partridge, A. H., Fann, J. R., Wolpin, S., ... & Ford, R. (2014) |
| 30 | Kamalumpundi et al. (2022) | Boele, F. W., Klein, M., Verdonck-de Leeuw, I. M., Cuijpers, P., Heimans, J. J., Snijders, T. J., ... & Reijneveld, J. C. (2018) |
| 31 | Singleton et al. (2022) | Børøsund, E., Cvancarova, M., Moore, S. M., Ekstedt, M., & Ruland, C. M. (2014) |
| 32 | Zhang et al. (2022) | Børøsund, E., Cvancarova, M., Moore, S. M., Ekstedt, M., & Ruland, C. M. (2014) |
| 33 | Buneviciene et al. (2021) | Børøsund, E., Varsi, C., Clark, M. M., Ehlers, S. L., Andrykowski, M. A., Sleveland, H. R. S., ... & Nes, L. S. (2020) |
| 34 | Wan et al. (2022) | Børøsund, E., Varsi, C., Clark, M. M., Ehlers, S. L., Andrykowski, M. A., Sleveland, H. R. S., ... & Nes, L. S. (2020) |
| 35 | Kamalumpundi et al. (2022) | Bouma, G., de Hosson, L. D., van Woerkom, C. E., van Essen, H., de Bock, G. H., Admiraal, J. M., ... & Walenkamp, A. M. (2017) |
| 36 | Kim et al. (2019) | Bray, V. J., Dhillon, H. M., Bell, M. L., Kabourakis, M., Fiero, M. H., Yip, D., ... & Vardy, J. L. (2017) |
| 37 | Golita et al. (2019) | Bruggeman-Everts, F. Z., Wolvers, M. D., Van de Schoot, R., Vollenbroek-Hutten, M. M., & Van der Lee, M. L. (2017) |
| 38 | Ester et al. (2021) | Buscemi, J., Oswald, L. B., Baik, S. H., Buitrago, D., Iacobelli, F., Phillips, S. M., ... & Yanez, B. (2020) |
| 39 | Wang et al. (2022) | Cadmus-Bertram, L., Tevaarwerk, A. J., Sesto, M. E., Gangnon, R., & Van Remortel, B. (2019) |
| 40 | Ester et al. (2021) | Cairo, J., Williams, L., Bray, L., Goetzke, K., & Perez, A. C. (2020) |
| 41 | Golita et al. (2019) | Carpenter, K. M., Stoner, S. A., Schmitz, K., McGregor, B. A., & Doorenbos, A. Z. (2014) |
| 42 | Singleton et al. (2022) | Carpenter, K. M., Stoner, S. A., Schmitz, K., McGregor, B. A., & Doorenbos, A. Z. (2014) |
| 43 | Golita et al. (2019) | Chambers, S. K., Ritterband, L. M., Thorndike, F., Nielsen, L., Aitken, J. F., Clutton, S., ... & Dunn, J. (2018) |
| 44 | Wan et al. (2022) | Chambers, S. K., Ritterband, L. M., Thorndike, F., Nielsen, L., Aitken, J. F., Clutton, S., ... & Dunn, J. (2018) |
| 45 | Ester et al. (2021) | Chan, J. M., Van Blarigan, E. L., Langlais, C. S., Zhao, S., Ramsdill, J. W., Daniel, K., ... & Winters-Stone, K. M. (2020) |
| 46 | Wang et al. (2022) | Chan, J. M., Van Blarigan, E. L., Langlais, C. S., Zhao, S., Ramsdill, J. W., Daniel, K., ... & Winters-Stone, K. M. (2020) |
| 47 | Dorri et al. (2019) | Chapman, J., Fletcher, C., Flight, I., & Wilson, C. (2018) |
| 48 | Ester et al. (2021) | Chapman, J., Fletcher, C., Flight, I., & Wilson, C. (2018) |
| 49 | Singleton et al. (2022) | Chee, W., Lee, Y., Im, E. O., Chee, E., Tsai, H. M., Nishigaki, M., ... & Mao, J. J. (2017) |
| 50 | Huang et al. (2019) | Chen, JX. (2018) |
| 51 | Buneviciene et al. (2021) | Cheong, I. Y., An, S. Y., Cha, W. C., Rha, M. Y., Kim, S. T., Chang, D. K., & Hwang, J. H. (2018) |
| 52 | Ester et al. (2021) | Cheong, I. Y., An, S. Y., Cha, W. C., Rha, M. Y., Kim, S. T., Chang, D. K., & Hwang, J. H. (2018) |
| 53 | Kaltenbaugh et al. (2015) | Chih, M. Y., DuBenske, L. L., Hawkins, R. P., Brown, R. L., Dinauer, S. K., Cleary, J. F., & Gustafson, D. H. (2013) |
| 54 | Ester et al. (2021) | Chow, E. J., Doody, D. R., Di, C., Armenian, S. H., Baker, K. S., Bricker, J. B., ... & Mendoza, J. A. (2021) |
| 55 | Wang et al. (2022) | Chow, E. J., Doody, D. R., Di, C., Armenian, S. H., Baker, K. S., Bricker, J. B., ... & Mendoza, J. A. (2021) |
| 56 | Ester et al. (2021) | Chung, I. Y., Jung, M., Park, Y. R., Cho, D., Chung, H., Min, Y. H., ... & Lee, J. W. (2020) |
| 57 | Kang et al. (2018) | Classen, C. C., Chivers, M. L., Urowitz, S., Barbera, L., Wiljer, D., O'Rinn, S., & Ferguson, S. E. (2013) |
| 58 | McAlpine et al. (2015) | Cleeland, C. S., Wang, X. S., Shi, Q., Mendoza, T. R., Wright, S. L., Berry, M. D., ... & Vaporciyan, A. A. (2011) |
| 59 | Kamalumpundi et al. (2022) | Cockle-Hearne, J., Barnett, D., Hicks, J., Simpson, M., White, I., & Faithfull, S. (2018) |
| 60 | Qan'ir et al. (2019) | Cockle-Hearne, J., Barnett, D., Hicks, J., Simpson, M., White, I., & Faithfull, S. (2018) |
| 61 | Buneviciene et al. (2021) | Compen, F. R., Bisseling, E. M., Schellekens, M. P. J., Donders, R., Carlson, L., Lee, M., & Speckens, A. E. M. (2018) |
| 62 | Golita et al. (2019) | Compen, F. R., Bisseling, E. M., Schellekens, M. P. J., Donders, R., Carlson, L., Lee, M., & Speckens, A. E. M. (2018) |
| 63 | Ramsey et al. (2020) | Conklin, H. M., Ogg, R. J., Ashford, J. M., Scoggins, M. A., Zou, P., Clark, K. N., ... & Zhang, H. (2015) |
| 64 | Seiler et al. (2017) | Corbett, T., Walsh, J. C., Groarke, A., Moss-Morris, R., & McGuire, B. E. (2016) |
| 65 | Ester et al. (2021) | Cox, M., Basen-Engquist, K., Carmack, C. L., Blalock, J., Li, Y., Murray, J., ... & Demark-Wahnefried, W. (2017) |
| 66 | Kim et al. (2019) | Damholdt, M. F., Mehlsen, M., O'Toole, M. S., Andreasen, R. K., Pedersen, A. D., & Zachariae, R. (2016) |
| 67 | Kamalumpundi et al. (2022) | David, N., Schlenker, P., Prudlo, U., & Larbig, W. (2011) |
| 68 | Golita et al. (2019) | David, N., Schlenker, P., Prudlo, U., & Larbig, W. (2013) |
| 69 | Huang et al. (2019) | Ding JX, Wang T, Wang WL, et al. (2016) |
| 70 | Wang et al. (2022) | Dong, X., Yi, X., Gao, D., Gao, Z., Huang, S., Chao, M., ... & Ding, M. (2019) |
| 71 | Kamalumpundi et al. (2022) | Donovan, H. S., Ward, S. E., Sereika, S. M., Knapp, J. E., Sherwood, P. R., Bender, C. M., ... & Ingel, R. (2014) |
| 72 | Zheng et al. (2020) | Dorfman, C. S., Kelleher, S. A., Winger, J. G., Shelby, R. A., Thorn, B. E., Sutton, L. M., ... & Somers, T. J. (2019) |
| 73 | Wan et al. (2022) | Dragomanovich, H. M., Dhruva, A., Ekman, E., Schoenbeck, K. L., Kubo, A., Van Blarigan, E. L., ... & Atreya, C. E. (2021) |
| 74 | Kaltenbaugh et al. (2015) | DuBenske, L. L., Gustafson, D. H., Shaw, B. R., & Cleary, J. F. (2010) |
| 75 | Wan et al. (2022) | Duffecy, J., Sanford, S., Wagner, L., Begale, M., Nawacki, E., & Mohr, D. C. (2013) |
| 76 | Ester et al. (2021) | Edbrooke, L., Granger, C. L., Clark, R. A., & Denehy, L. (2019) |
| 77 | Ramsey et al. (2020) | Emmons, K. M., Puleo, E., Sprunck-Harrild, K., Ford, J., Ostroff, J. S., Hodgson, D., ... & Tyc, V. (2013) |
| 78 | Seiler et al. (2017) | Everts, F. Z. B., Van Der Lee, M. L., & de Jager Meezenbroek, E. (2015) |
| 79 | Singleton et al. (2022) | Fang, S. Y., Wang, Y. L., Lu, W. H., Lee, K. T., Kuo, Y. L., & Fetzer, S. J. (2020) |
| 80 | Kamalumpundi et al. (2022) | Fann, J. R., Hong, F., Halpenny, B., Blonquist, T. M., & Berry, D. L. (2017) |
| 81 | Zhang et al. (2022) | Fann, J. R., Hong, F., Halpenny, B., Blonquist, T. M., & Berry, D. L. (2017) |
| 82 | Kaltenbaugh et al. (2015) | Farnham, S., Cheng, L., Stone, L., Zaner-Godsey, M., Hibbeln, C., Syrjala, K., ... & Abrams, J. (2002, April) |
| 83 | Ramsey et al. (2020) | Fazelniya, Z., Najafi, M., Moafi, A., & Talakoub, S. (2017) |
| 84 | Ester et al. (2021) | Fazzino, T. L., Fabian, C., & Befort, C. A. (2017) |
| 85 | Buneviciene et al. (2021) | Ferrante, J. M., Devine, K. A., Bator, A., Rodgers, A., Ohman-Strickland, P. A., Bandera, E. V., & Hwang, K. O. (2020) |
| 86 | Singleton et al. (2022) | Ferrante, J. M., Devine, K. A., Bator, A., Rodgers, A., Ohman-Strickland, P. A., Bandera, E. V., & Hwang, K. O. (2020) |
| 87 | Wang et al. (2022) | Ferrante, J. M., Devine, K. A., Bator, A., Rodgers, A., Ohman-Strickland, P. A., Bandera, E. V., & Hwang, K. O. (2020) |
| 88 | Wang et al. (2022) | Finlay, A., Evans, H., Vincent, A., Wittert, G., Vandelanotte, C., & Short, C. E. (2020) |
| 89 | Singleton et al. (2022) | Fjell, M., Langius-Eklöf, A., Nilsson, M., Wengström, Y., & Sundberg, K. (2020) |
| 90 | Dorri et al. (2019) | Forbes, C. C., Blanchard, C. M., Mummery, W. K., & Courneya, K. S. (2015) |
| 91 | Ester et al. (2021) | Forbes, C. C., Blanchard, C. M., Mummery, W. K., & Courneya, K. S. (2015) |
| 92 | Kiss et al. (2019) | Forbes, C. C., Blanchard, C. M., Mummery, W. K., & Courneya, K. S. (2015) |
| 93 | Seiler et al. (2017) | Foster, C., Grimmett, C., May, C. M., Ewings, S., Myall, M., Hulme, C., ... & Richardson, A. (2016) |
| 94 | Seiler et al. (2017) | Foster, C., Grimmett, C., May, C. M., Ewings, S., Myall, M., Hulme, C., ... & Richardson, A. (2016) |
| 95 | Kamalumpundi et al. (2022) | Fox, R. S., Moreno, P. I., Yanez, B., Estabrook, R., Thomas, J., Bouchard, L. C., ... & Penedo, F. J. (2019) |
| 96 | Seiler et al. (2017) | Freeman, L. W., White, R., Ratcliff, C. G., Sutton, S., Stewart, M., Palmer, J. L., ... & Cohen, L. (2015) |
| 97 | Singleton et al. (2022) | Freeman, L. W., White, R., Ratcliff, C. G., Sutton, S., Stewart, M., Palmer, J. L., ... & Cohen, L. (2015) |
| 98 | Buneviciene et al. (2021) | Frensham, L. J., Parfitt, G., & Dollman, J. (2018) |
| 99 | Ester et al. (2021) | Frensham, L. J., Parfitt, G., & Dollman, J. (2018) |
| 100 | Ester et al. (2021) | Frensham, L. J., Parfitt, G., & Dollman, J. (2020) |
| 101 | Kiss et al. (2019) | Galiano-Castillo, N., Arroyo-Morales, M., Lozano-Lozano, M., Fernández-Lao, C., Martín-Martín, L., Del-Moral-Ávila, R., & Cantarero-Villanueva, I. (2017) |
| 102 | Singleton et al. (2022) | Galiano-Castillo, N., Arroyo-Morales, M., Lozano-Lozano, M., Fernández-Lao, C., Martín-Martín, L., Del-Moral-Ávila, R., & Cantarero-Villanueva, I. (2017) |
| 103 | Buneviciene et al. (2021) | Galiano‐Castillo, N., Cantarero‐Villanueva, I., Fernández‐Lao, C., Ariza‐García, A., Díaz‐Rodríguez, L., Del‐Moral‐Ávila, R., & Arroyo‐Morales, M. (2016) |
| 104 | Kiss et al. (2019) | Galiano‐Castillo, N., Cantarero‐Villanueva, I., Fernández‐Lao, C., Ariza‐García, A., Díaz‐Rodríguez, L., Del‐Moral‐Ávila, R., & Arroyo‐Morales, M. (2016) |
| 105 | Seiler et al. (2017) | Galiano‐Castillo, N., Cantarero‐Villanueva, I., Fernández‐Lao, C., Ariza‐García, A., Díaz‐Rodríguez, L., Del‐Moral‐Ávila, R., & Arroyo‐Morales, M. (2016) |
| 106 | Singleton et al. (2022) | Galiano‐Castillo, N., Cantarero‐Villanueva, I., Fernández‐Lao, C., Ariza‐García, A., Díaz‐Rodríguez, L., Del‐Moral‐Ávila, R., & Arroyo‐Morales, M. (2016) |
| 107 | Ester et al. (2021) | Gehring, K., Kloek, C. J., Aaronson, N. K., Janssen, K. W., Jones, L. W., Sitskoorn, M. M., & Stuiver, M. M. (2018) |
| 108 | Ester et al. (2021) | Gell, N. M., Grover, K. W., Humble, M., Sexton, M., & Dittus, K. (2017) |
| 109 | Ester et al. (2021) | Gell, N. M., Grover, K. W., Savard, L., & Dittus, K. (2020) |
| 110 | Wang et al. (2022) | Gell, N. M., Grover, K. W., Savard, L., & Dittus, K. (2020) |
| 111 | Ramsey et al. (2020) | Gershon, J., Zimand, E., Pickering, M., Rothbaum, B. O., & Hodges, L. (2004) |
| 112 | Wan et al. (2022) | Giesler, J. M., Keller, B., Repke, T., Leonhart, R., Weis, J., Muckelbauer, R., ... & Holmberg, C. (2017) |
| 113 | Hong et al. (2021) | Gilliam, M. B., Ross, K., Futch, L., Walsh, A., Klapow, J., Davis, D., ... & Madan-Swain, A. (2011) |
| 114 | Ramsey et al. (2020) | Gilliam, M. B., Ross, K., Futch, L., Walsh, A., Klapow, J., Davis, D., ... & Madan-Swain, A. (2011) |
| 115 | Kiss et al. (2019) | Gnagnarella, P., Misotti, A. M., Santoro, L., Akoumianakis, D., Del Campo, L., De Lorenzo, F., ... & McVie, J. G. (2016) |
| 116 | Wang et al. (2022) | Gnagnarella, P., Misotti, A. M., Santoro, L., Akoumianakis, D., Del Campo, L., De Lorenzo, F., ... & McVie, J. G. (2016) |
| 117 | Ester et al. (2021) | Gokal, K., Wallis, D., Ahmed, S., Boiangiu, I., Kancherla, K., & Munir, F. (2016) |
| 118 | Ester et al. (2021) | Golsteijn, R. H. J., Bolman, C., Volders, E., Peels, D. A., de Vries, H., & Lechner, L. (2018) |
| 119 | Wang et al. (2022) | Golsteijn, R. H. J., Bolman, C., Volders, E., Peels, D. A., de Vries, H., & Lechner, L. (2018) |
| 120 | Ester et al. (2021) | Gomersall, S. R., Skinner, T. L., Winkler, E., Healy, G. N., Eakin, E., & Fjeldsoe, B. (2019) |
| 121 | Wang et al. (2022) | Gomersall, S. R., Skinner, T. L., Winkler, E., Healy, G. N., Eakin, E., & Fjeldsoe, B. (2019) |
| 122 | Buneviciene et al. (2021) | Graetz, I., Anderson, J. N., McKillop, C. N., Stepanski, E. J., Paladino, A. J., & Tillmanns, T. D. (2018) |
| 123 | Buneviciene et al. (2021) | Greer, J. A., Jacobs, J., Pensak, N., MacDonald, J. J., Fuh, C. X., Perez, G. K., ... & Temel, J. S. (2019) |
| 124 | Kamalumpundi et al. (2022) | Greer, J. A., Jacobs, J., Pensak, N., MacDonald, J. J., Fuh, C. X., Perez, G. K., ... & Temel, J. S. (2019) |
| 125 | Seiler et al. (2017) | Grimmett, C., Armes, J., Breckons, M., Calman, L., Corner, J., Fenlon, D., ... & Foster, C. (2013) |
| 126 | Ester et al. (2021) | Groen, W. G., Kuijpers, W., Oldenburg, H. S., Wouters, M. W., Aaronson, N. K., & van Harten, W. H. (2017) |
| 127 | McAlpine et al. (2015) | Gustafson, D. H., Hawkins, R., McTavish, F., Pingree, S., Chen, W. C., Volrathongchai, K., ... & Serlin, R. C. (2008) |
| 128 | Singleton et al. (2022) | Gustafson, D. H., Hawkins, R., McTavish, F., Pingree, S., Chen, W. C., Volrathongchai, K., ... & Serlin, R. C. (2008) |
| 129 | Ester et al. (2021) | Haggerty, A. F., Hagemann, A., Barnett, M., Thornquist, M., Neuhouser, M. L., Horowitz, N., ... & Allison, K. C. (2017) |
| 130 | Buneviciene et al. (2021) | Ham, K., Chin, S., Suh, Y. J., Rhee, M., Yu, E. S., Lee, H. J., ... & Chung, K. M. (2019) |
| 131 | Kamalumpundi et al. (2022) | Ham, K., Chin, S., Suh, Y. J., Rhee, M., Yu, E. S., Lee, H. J., ... & Chung, K. M. (2019) |
| 132 | Singleton et al. (2022) | Handa, S., Okuyama, H., Yamamoto, H., Nakamura, S., & Kato, Y. (2020) |
| 133 | Hong et al. (2021) | Hardy, K. K., Willard, V. W., & Bonner, M. J. (2011) |
| 134 | Ramsey et al. (2020) | Hardy, K. K., Willard, V. W., Allen, T. M., & Bonner, M. J. (2013) |
| 135 | Dorri et al. (2019) | Hartman, S. J., Nelson, S. H., & Weiner, L. S. (2018) |
| 136 | Ester et al. (2021) | Hartman, S. J., Nelson, S. H., & Weiner, L. S. (2018) |
| 137 | Dorri et al. (2019) | Hatchett, A., Hallam, J. S., & Ford, M. A. (2013) |
| 138 | Ester et al. (2021) | Hatchett, A., Hallam, J. S., & Ford, M. A. (2013) |
| 139 | Haberlin et al. (2018) | Hatchett, A., Hallam, J. S., & Ford, M. A. (2013) |
| 140 | Wan et al. (2022) | Hauffman, A., Alfonsson, S., Bill‐Axelson, A., Bergkvist, L., Forslund, M., Mattsson, S., ... & Johansson, B. (2020) |
| 141 | Singleton et al. (2022) | Hawkins, R. P., Pingree, S., Baker, T. B., Roberts, L. J., Shaw, B. R., McDowell, H., ... & Gustafson, D. H. (2011) |
| 142 | Singleton et al. (2022) | Hawkins, R. P., Pingree, S., Shaw, B., Serlin, R. C., Swoboda, C., Han, J. Y., ... & Salner, A. (2010) |
| 143 | Qan'ir et al. (2019) | Hawkins, R. P., Pingree, S., Van Bogaert, D., McDowell, H., Jarrard, D., Carmack, C., & Salner, A. (2017) |
| 144 | Ester et al. (2021) | Hong, Y. A., Goldberg, D., Ory, M. G., Towne Jr, S. D., Forjuoh, S. N., Kellstedt, D., & Wang, S. (2015) |
| 145 | Haberlin et al. (2018) | Hong, Y. A., Goldberg, D., Ory, M. G., Towne Jr, S. D., Forjuoh, S. N., Kellstedt, D., & Wang, S. (2015) |
| 146 | Haberlin et al. (2018) | Hooke, M. C., Gilchrist, L., Tanner, L., Hart, N., & Withycombe, J. S. (2016) |
| 147 | Ramsey et al. (2020) | Hooke, M. C., Gilchrist, L., Tanner, L., Hart, N., & Withycombe, J. S. (2016) |
| 148 | Singleton et al. (2022) | Hou, I. C., Lin, H. Y., Shen, S. H., Chang, K. J., Tai, H. C., Tsai, A. J., & Dykes, P. C. (2020) |
| 149 | Zhang et al. (2022) | Hou, I. C., Lin, H. Y., Shen, S. H., Chang, K. J., Tai, H. C., Tsai, A. J., & Dykes, P. C. (2020) |
| 150 | Hong et al. (2021) | Howell, C. R., Krull, K. R., Partin, R. E., Kadan‐Lottick, N. S., Robison, L. L., Hudson, M. M., & Ness, K. K. (2018) |
| 151 | Ramsey et al. (2020) | Huang, J. S., Dillon, L., Terrones, L., Schubert, L., Roberts, W., Finklestein, J., ... & Patrick, K. (2014) |
| 152 | Wan et al. (2022) | Huang, Q., Zhuang, Y., Ye, X., Li, M., Liu, Z., Li, J., & Pan, Z. (2021) |
| 153 | Golita et al. (2019) | Hummel, S. B., van Lankveld, J. J., Oldenburg, H. S., Hahn, D. E., Kieffer, J. M., Gerritsma, M. A., ... & Aaronson, N. K. (2017) |
| 154 | Singleton et al. (2022) | Hummel, S. B., van Lankveld, J. J., Oldenburg, H. S., Hahn, D. E., Kieffer, J. M., Gerritsma, M. A., ... & Aaronson, N. K. (2017) |
| 155 | Kamalumpundi et al. (2022) | Im, E. O., Kim, S., Lee, C., Chee, E., Mao, J. J., & Chee, W. (2019) |
| 156 | Ester et al. (2021) | Javaheri, P. A., Nekolaichuk, C., Haennel, R., Parliament, M. B., & McNeely, M. L. (2015) |
| 157 | Buneviciene et al. (2021) | Ji, W., Kwon, H., Lee, S., Kim, S., Hong, J. S., Park, Y. R., ... & Choi, C. M. (2019) |
| 158 | Ramsey et al. (2020) | Jibb, L. A., Stevens, B. J., Nathan, P. C., Seto, E., Cafazzo, J. A., Johnston, D. L., ... & Stinson, J. N. (2017) |
| 159 | Zheng et al. (2020) | Jibb, L. A., Stevens, B. J., Nathan, P. C., Seto, E., Cafazzo, J. A., Johnston, D. L., ... & Stinson, J. N. (2017) |
| 160 | Dorri et al. (2019) | Kanera, I. M., Bolman, C. A., Willems, R. A., Mesters, I., & Lechner, L. (2016) |
| 161 | Ester et al. (2021) | Kanera, I. M., Bolman, C. A., Willems, R. A., Mesters, I., & Lechner, L. (2016) |
| 162 | Kiss et al. (2019) | Kanera, I. M., Bolman, C. A., Willems, R. A., Mesters, I., & Lechner, L. (2016) |
| 163 | Ester et al. (2021) | Kanera, I. M., Willems, R. A., Bolman, C. A., Mesters, I., Verboon, P., & Lechner, L. (2017) |
| 164 | Haberlin et al. (2018) | Kanera, I. M., Willems, R. A., Bolman, C. A., Mesters, I., Verboon, P., & Lechner, L. (2017) |
| 165 | Kiss et al. (2019) | Kanera, I. M., Willems, R. A., Bolman, C. A., Mesters, I., Verboon, P., & Lechner, L. (2017) |
| 166 | Ramsey et al. (2020) | Kato, P. M., Cole, S. W., Bradlyn, A. S., & Pollock, B. H. (2008) |
| 167 | Kamalumpundi et al. (2022) | Kelleher, S. A., Winger, J. G., Dorfman, C. S., Ingle, K. K., Moskovich, A. A., Abernethy, A. P., ... & Somers, T. J. (2019) |
| 168 | Ester et al. (2021) | Kenfield, S. A., Van Blarigan, E. L., Ameli, N., Lavaki, E., Cedars, B., Paciorek, A. T., ... & Chan, J. M. (2019) |
| 169 | Wang et al. (2022) | Kenfield, S. A., Van Blarigan, E. L., Ameli, N., Lavaki, E., Cedars, B., Paciorek, A. T., ... & Chan, J. M. (2019) |
| 170 | Ramsey et al. (2020) | Kesler, S. R., Lacayo, N. J., & Jo, B. (2011) |
| 171 | Kim et al. (2019) | Kesler, S., Hosseini, S. H., Heckler, C., Janelsins, M., Palesh, O., Mustian, K., & Morrow, G. (2013) |
| 172 | Kamalumpundi et al. (2022) | Kim, B. Y., Park, K. J., & Ryoo, S. B. (2018) |
| 173 | Wan et al. (2022) | Kim, B. Y., Park, K. J., & Ryoo, S. B. (2018) |
| 174 | Singleton et al. (2022) | Kim, H. J., Kim, S. M., Shin, H., Jang, J. S., Kim, Y. I., & Han, D. H. (2018) |
| 175 | Singleton et al. (2022) | Kim, S. C., Hawkins, R. P., Shah, D. V., Gustafson, D. H., & Baker, T. B. (2020) |
| 176 | Singleton et al. (2022) | Kim, S. C., Shaw, B. R., Shah, D. V., Hawkins, R. P., Pingree, S., McTavish, F. M., & Gustafson, D. H. (2019) |
| 177 | Kamalumpundi et al. (2022) | Kinner, E. M., Armer, J. S., McGregor, B. A., Duffecy, J., Leighton, S., Corden, M. E., ... & Lutgendorf, S. K. (2018) |
| 178 | Kamalumpundi et al. (2022) | Knoerl, R., Barton, D. L., Holden, J. E., Krauss, J. C., LaVasseur, B., & Smith, E. M. (2018) |
| 179 | Zhang et al. (2022) | Köhle, N., Drossaert, C. H., Ten Klooster, P. M., Schreurs, K. M., Hagedoorn, M., Uden-Kraan, V., ... & Bohlmeijer, E. T. (2021) |
| 180 | McAlpine et al. (2015) | Korda, H., & Itani, Z. (2013) |
| 181 | Kiss et al. (2019) | Krebs, P., Shtaynberger, J., McCabe, M., Iocolano, M., Williams, K., Shuk, E., & Ostroff, J. S. (2017) |
| 182 | Buneviciene et al. (2021) | Kubo, A., Kurtovich, E., McGinnis, M., Aghaee, S., Altschuler, A., Quesenberry Jr, C., ... & Avins, A. L. (2019) |
| 183 | Dorri et al. (2019) | Kuijpers W, Groen WG, Oldenburg HS, Wouters MW, Aaronson NK, van Harten WH. (2016) |
| 184 | Ramsey et al. (2020) | Kunin‐Batson, A., Steele, J., Mertens, A., & Neglia, J. P. (2016) |
| 185 | Singleton et al. (2022) | Lally, R. M., Kupzyk, K. A., Bellavia, G., Hydeman, J., Gallo, S., Helgeson, V. S., ... & Brown, J. K. (2020) |
| 186 | Qan'ir et al. (2019) | Lange, L., Fink, J., Bleich, C., Graefen, M., & Schulz, H. (2017) |
| 187 | Hong et al. (2021) | Le, A., Mitchell, H. R., Zheng, D. J., Rotatori, J., Fahey, J. T., Ness, K. K., & Kadan‐Lottick, N. S. (2017) |
| 188 | Ramsey et al. (2020) | Le, A., Mitchell, H. R., Zheng, D. J., Rotatori, J., Fahey, J. T., Ness, K. K., & Kadan‐Lottick, N. S. (2017) |
| 189 | Dorri et al. (2019) | Lee, M. K., Yun, Y. H., Park, H. A., Lee, E. S., Jung, K. H., & Noh, D. Y. (2014) |
| 190 | Haberlin et al. (2018) | Lee, M. K., Yun, Y. H., Park, H. A., Lee, E. S., Jung, K. H., & Noh, D. Y. (2014) |
| 191 | Kiss et al. (2019) | Lee, M. K., Yun, Y. H., Park, H. A., Lee, E. S., Jung, K. H., & Noh, D. Y. (2014) |
| 192 | Zheng et al. (2020) | Lengacher, C. A., Reich, R. R., Ramesar, S., Alinat, C. B., Moscoso, M., Cousin, L., ... & Park, J. Y. (2018) |
| 193 | Huang et al. (2019) | Li YL, Wang G, Wang MH, et al. (2005) |
| 194 | Ramsey et al. (2020) | Li, W. H., Chung, J. O., & Ho, E. K. (2011) |
| 195 | McAlpine et al. (2015) | Lieberman, M. A., & Goldstein, B. A. (2005) |
| 196 | McAlpine et al. (2015) | Lieberman, M. A., Golant, M., Giese‐Davis, J., Winzlenberg, A., Benjamin, H., Humphreys, K., ... & Spiegel, D. (2003) |
| 197 | Ester et al. (2021) | Linda Abbott, D. N. P. (2017) |
| 198 | Huang et al. (2019) | Liu JP, Liu YH, Hu DW, et al. (2016) |
| 199 | Qan'ir et al. (2019) | Loiselle, C. G., Edgar, L., Batist, G., Lu, J., & Lauzier, S. (2010) |
| 200 | Buneviciene et al. (2021) | Lozano-Lozano, M., Cantarero-Villanueva, I., Martin-Martin, L., Galiano-Castillo, N., Sanchez, M. J., Fernández-Lao, C., ... & Arroyo-Morales, M. (2019) |
| 201 | Huang et al. (2019) | Luo, J. (2012) |
| 202 | Ester et al. (2021) | Lynch, B. M., Nguyen, N. H., Moore, M. M., Reeves, M. M., Rosenberg, D. E., Boyle, T., ... & English, D. R. (2019) |
| 203 | Singleton et al. (2022) | Lynch, B. M., Nguyen, N. H., Moore, M. M., Reeves, M. M., Rosenberg, D. E., Boyle, T., ... & English, D. R. (2019) |
| 204 | Ester et al. (2021) | Lynch, B. M., Nguyen, N. H., Moore, M. M., Reeves, M. M., Rosenberg, D. E., Boyle, T., ... & English, D. R. (2019) |
| 205 | Ester et al. (2021) | MacDonald, A. M., Chafranskaia, A., Lopez, C. J., Maganti, M., Bernstein, L. J., Chang, E., ... & Jones, J. M. (2020) |
| 206 | Ester et al. (2021) | Maxwell‐Smith, C., Hince, D., Cohen, P. A., Bulsara, M. K., Boyle, T., Platell, C., ... & Hardcastle, S. J. (2019) |
| 207 | Wang et al. (2022) | Maxwell‐Smith, C., Hince, D., Cohen, P. A., Bulsara, M. K., Boyle, T., Platell, C., ... & Hardcastle, S. J. (2019) |
| 208 | Ester et al. (2021) | Mayer, D. K., Landucci, G., Awoyinka, L., Atwood, A. K., Carmack, C. L., Demark-Wahnefried, W., ... & Gustafson, D. H. (2018) |
| 209 | Kiss et al. (2019) | Mayer, D. K., Landucci, G., Awoyinka, L., Atwood, A. K., Carmack, C. L., Demark-Wahnefried, W., ... & Gustafson, D. H. (2018) |
| 210 | Wang et al. (2022) | Mayer, D. K., Landucci, G., Awoyinka, L., Atwood, A. K., Carmack, C. L., Demark-Wahnefried, W., ... & Gustafson, D. H. (2018) |
| 211 | Ester et al. (2021) | Mayo, N. E., Moriello, C., Scott, S. C., Dawes, D., Auais, M., & Chasen, M. (2014) |
| 212 | Buneviciene et al. (2021) | McCarroll, M. L., Armbruster, S., Pohle-Krauza, R. J., Lyzen, A. M., Min, S., Nash, D. W., ... & von Gruenigen, V. E. (2015) |
| 213 | Dorri et al. (2019) | McCarroll, M. L., Armbruster, S., Pohle-Krauza, R. J., Lyzen, A. M., Min, S., Nash, D. W., ... & von Gruenigen, V. E. (2015) |
| 214 | Ester et al. (2021) | McCarroll, M. L., Armbruster, S., Pohle-Krauza, R. J., Lyzen, A. M., Min, S., Nash, D. W., ... & von Gruenigen, V. E. (2015) |
| 215 | Haberlin et al. (2018) | McCarroll, M. L., Armbruster, S., Pohle-Krauza, R. J., Lyzen, A. M., Min, S., Nash, D. W., ... & von Gruenigen, V. E. (2015) |
| 216 | Buneviciene et al. (2021) | McCarthy, M. S., Matthews, E. E., Battaglia, C., & Meek, P. M. (2018, September) |
| 217 | Ester et al. (2021) | McNeil, J., Brenner, D. R., Stone, C. R., O’Reilly, R., Ruan, Y., Vallance, J. K., ... & Friedenreich, C. M. (2019) |
| 218 | Wang et al. (2022) | McNeil, J., Brenner, D. R., Stone, C. R., O’Reilly, R., Ruan, Y., Vallance, J. K., ... & Friedenreich, C. M. (2019) |
| 219 | Wang et al. (2022) | McNeil, J., Fahim, M., Stone, C. R., O’Reilly, R., Courneya, K. S., & Friedenreich, C. M. (2022) |
| 220 | Buneviciene et al. (2021) | Mendoza, J. A., Baker, K. S., Moreno, M. A., Whitlock, K., Abbey‐Lambertz, M., Waite, A., ... & Chow, E. J. (2017) |
| 221 | Hong et al. (2021) | Mendoza, J. A., Baker, K. S., Moreno, M. A., Whitlock, K., Abbey‐Lambertz, M., Waite, A., ... & Chow, E. J. (2017) |
| 222 | Ramsey et al. (2020) | Mendoza, J. A., Baker, K. S., Moreno, M. A., Whitlock, K., Abbey‐Lambertz, M., Waite, A., ... & Chow, E. J. (2017) |
| 223 | McAlpine et al. (2015) | Meneses, K., McNees, P., Azuero, A., & Jukkala, A. (2010) |
| 224 | Kim et al. (2019) | Mihuta, M. E., Green, H. J., & Shum, D. H. (2018) |
| 225 | Wang et al. (2022) | Mohamad, H., Ntessalen, M., Craig, L. C. A., Clark, J., Fielding, S., N’Dow, J., ... & McNeill, G. (2019) |
| 226 | Wan et al. (2022) | Murphy, M. J., Newby, J. M., Butow, P., Loughnan, S. A., Joubert, A. E., Kirsten, L., ... & Andrews, G. (2020) |
| 227 | Ester et al. (2021) | Naito, T., Mitsunaga, S., Miura, S., Tatematsu, N., Inano, T., Mouri, T., ... & Takayama, K. (2019) |
| 228 | Kaltenbaugh et al. (2015) | Namkoong, K., DuBenske, L. L., Shaw, B. R., Gustafson, D. H., Hawkins, R. P., Shah, D. V., ... & Cleary, J. F. (2012) |
| 229 | Ester et al. (2021) | Nápoles, A. M., Santoyo-Olsson, J., Chacón, L., Stewart, A. L., Dixit, N., & Ortiz, C. (2019) |
| 230 | Wang et al. (2022) | Nguyen, N. H., Vallance, J. K., Buman, M. P., Moore, M. M., Reeves, M. M., Rosenberg, D. E., ... & Lynch, B. M. (2021) |
| 231 | Kamalumpundi et al. (2022) | Northouse, L., Schafenacker, A., Barr, K. L., Katapodi, M., Yoon, H., Brittain, K., ... & An, L. (2014) |
| 232 | Wan et al. (2022) | Northouse, L., Schafenacker, A., Barr, K. L., Katapodi, M., Yoon, H., Brittain, K., ... & An, L. (2014) |
| 233 | Ester et al. (2021) | Nyrop, K. A., Deal, A. M., Choi, S. K., Wagoner, C. W., Lee, J. T., Wood, A., ... & Muss, H. B. (2018) |
| 234 | Zheng et al. (2020) | Oldenmenger, W. H., Baan, M. A., & van der Rijt, C. C. (2018) |
| 235 | Ester et al. (2021) | Ormel, H. L., van der Schoot, G. G., Westerink, N. D. L., Sluiter, W. J., Gietema, J. A., & Walenkamp, A. M. (2018) |
| 236 | Kiss et al. (2019) | Ormel, H. L., van der Schoot, G. G., Westerink, N. D. L., Sluiter, W. J., Gietema, J. A., & Walenkamp, A. M. (2018) |
| 237 | Wang et al. (2022) | Ormel, H. L., van der Schoot, G. G., Westerink, N. D. L., Sluiter, W. J., Gietema, J. A., & Walenkamp, A. M. (2018) |
| 238 | McAlpine et al. (2015) | Osei, D. K., Lee, J. W., Modest, N. N., & Pothier, P. K. (2013) |
| 239 | Qan'ir et al. (2019) | Osei, D. K., Lee, J. W., Modest, N. N., & Pothier, P. K. (2013) |
| 240 | Golita et al. (2019) | Owen, J. E., Klapow, J. C., Roth, D. L., Shuster, J. L., Bellis, J., Meredith, R., & Tucker, D. C. (2005) |
| 241 | McAlpine et al. (2015) | Owen, J. E., Klapow, J. C., Roth, D. L., Shuster, J. L., Bellis, J., Meredith, R., & Tucker, D. C. (2005) |
| 242 | Singleton et al. (2022) | Owen, J. E., Klapow, J. C., Roth, D. L., Shuster, J. L., Bellis, J., Meredith, R., & Tucker, D. C. (2005) |
| 243 | Golita et al. (2019) | Owen, J. E., O'Carroll Bantum, E., Pagano, I. S., & Stanton, A. (2017) |
| 244 | Ramsey et al. (2020) | Palmer, S. L., Leigh, L., Ellison, S. C., Onar-Thomas, A., Wu, S., Qaddoumi, I., ... & Gajjar, A. (2014) |
| 245 | Buneviciene et al. (2021) | Pappot, H., Taarnhøj, G. A., Elsbernd, A., Hjerming, M., Hanghøj, S., Jensen, M., & Boisen, K. A. (2019) |
| 246 | Ester et al. (2021) | Park, J. H., Lee, J., Oh, M., Park, H., Chae, J., Kim, D. I., ... & Jeon, J. Y. (2015) |
| 247 | Ester et al. (2021) | Park, S. W., Lee, I., Kim, J. I., Park, H., Lee, J. D., Uhm, K. E., ... & Lee, J. Y. (2019) |
| 248 | Buneviciene et al. (2021) | Park, S., Kim, J. Y., Lee, J. C., Kim, H. R., Song, S., Kwon, H., ... & Choi, C. M. (2019) |
| 249 | Kamalumpundi et al. (2022) | Park, S., Kim, J. Y., Lee, J. C., Kim, H. R., Song, S., Kwon, H., ... & Choi, C. M. (2019) |
| 250 | Zheng et al. (2020) | Park, S., Kim, J. Y., Lee, J. C., Kim, H. R., Song, S., Kwon, H., ... & Choi, C. M. (2019) |
| 251 | Ester et al. (2021) | Park, S., Kim, K., Ahn, H. K., Kim, J. W., Min, G., Chung, B. H., & Koo, K. C. (2020) |
| 252 | Ester et al. (2021) | Paxton, R. J., Hajek, R., Newcomb, P., Dobhal, M., Borra, S., Taylor, W. C., ... & Jones, L. A. (2017) |
| 253 | Kamalumpundi et al. (2022) | Petzel, S. V., Isaksson Vogel, R., Cragg, J., McClellan, M., Chan, D., Jacko, J. A., ... & Geller, M. A. (2018) |
| 254 | Dorri et al. (2019) | Pope, Z. C., Zeng, N., Zhang, R., Lee, H. Y., & Gao, Z. (2018) |
| 255 | Ester et al. (2021) | Pope, Z. C., Zeng, N., Zhang, R., Lee, H. Y., & Gao, Z. (2018) |
| 256 | Kiss et al. (2019) | Pope, Z. C., Zeng, N., Zhang, R., Lee, H. Y., & Gao, Z. (2018) |
| 257 | Ester et al. (2021) | Pope, Z., Lee, J. E., Zeng, N., Lee, H. Y., & Gao, Z. (2019) |
| 258 | Kim et al. (2019) | Poppelreuter, M., Weis, J., & Bartsch, H. H. (2009) |
| 259 | Huang et al. (2019) | Purcell, A., Fleming, J., Burmeister, B., Bennett, S., & Haines, T. (2011) |
| 260 | Dorri et al. (2019) | Puszkiewicz, P., Roberts, A. L., Smith, L., Wardle, J., & Fisher, A. (2016) |
| 261 | Ester et al. (2021) | Puszkiewicz, P., Roberts, A. L., Smith, L., Wardle, J., & Fisher, A. (2016) |
| 262 | Dorri et al. (2019) | Quintiliani LM, Mann DM, Puputti M, Quinn E, Bowen DJ. (2016) |
| 263 | Dorri et al. (2019) | Rabin, C., Dunsiger, S., Ness, K. K., & Marcus, B. H. (2011) |
| 264 | Ester et al. (2021) | Rabin, C., Dunsiger, S., Ness, K. K., & Marcus, B. H. (2011) |
| 265 | Kiss et al. (2019) | Rabin, C., Dunsiger, S., Ness, K. K., & Marcus, B. H. (2011) |
| 266 | Seiler et al. (2017) | Rabin, C., Dunsiger, S., Ness, K. K., & Marcus, B. H. (2011) |
| 267 | Wan et al. (2022) | Rahimi, M., Mahdizadeh, M., Chamanzari, H., & Mahdizadeh, S. M. (2021) |
| 268 | Wang et al. (2022) | Rastogi, S., Tevaarwerk, A. J., Sesto, M., Van Remortel, B., Date, P., Gangnon, R., ... & Cadmus‐Bertram, L. (2020) |
| 269 | Golita et al. (2019) | Ritterband, L. M., Bailey, E. T., Thorndike, F. P., Lord, H. R., Farrell‐Carnahan, L., & Baum, L. D. (2012) |
| 270 | McAlpine et al. (2015) | Ritterband, L. M., Bailey, E. T., Thorndike, F. P., Lord, H. R., Farrell‐Carnahan, L., & Baum, L. D. (2012) |
| 271 | Seiler et al. (2017) | Ritterband, L. M., Bailey, E. T., Thorndike, F. P., Lord, H. R., Farrell‐Carnahan, L., & Baum, L. D. (2012) |
| 272 | Ester et al. (2021) | Robertson, M. C., Lyons, E. J., Liao, Y., Baum, M. L., & Basen-Engquist, K. M. (2020) |
| 273 | Buneviciene et al. (2021) | Rosen, K. D., Paniagua, S. M., Kazanis, W., Jones, S., & Potter, J. S. (2018) |
| 274 | Singleton et al. (2022) | Rosen, K. D., Paniagua, S. M., Kazanis, W., Jones, S., & Potter, J. S. (2018) |
| 275 | Kamalumpundi et al. (2022) | Ruland, C. M., Andersen, T., Jeneson, A., Moore, S., Grimsbø, G. H., Børøsund, E., & Ellison, M. C. (2013) |
| 276 | McAlpine et al. (2015) | Ruland, C. M., Andersen, T., Jeneson, A., Moore, S., Grimsbø, G. H., Børøsund, E., & Ellison, M. C. (2013) |
| 277 | Qan'ir et al. (2019) | Ruland, C. M., Andersen, T., Jeneson, A., Moore, S., Grimsbø, G. H., Børøsund, E., & Ellison, M. C. (2013) |
| 278 | Zhang et al. (2022) | Ruland, C. M., Andersen, T., Jeneson, A., Moore, S., Grimsbø, G. H., Børøsund, E., & Ellison, M. C. (2013) |
| 279 | Singleton et al. (2022) | Ryhänen, A. M., Rankinen, S., Siekkinen, M., Saarinen, M., Korvenranta, H., & Leino‐Kilpi, H. (2013) |
| 280 | Hong et al. (2021) | Sabel, M., Sjölund, A., Broeren, J., Arvidsson, D., Saury, J. M., Blomgren, K., ... & Emanuelson, I. (2016) |
| 281 | Ramsey et al. (2020) | Sabel, M., Sjölund, A., Broeren, J., Arvidsson, D., Saury, J. M., Blomgren, K., ... & Emanuelson, I. (2016) |
| 282 | Ramsey et al. (2020) | Sabel, M., Sjölund, A., Broeren, J., Arvidsson, D., Saury, J. M., Gillenstrand, J., ... & Lannering, B. (2017) |
| 283 | Kiss et al. (2019) | Sajid, S., Dale, W., Mustian, K., Kotwal, A., Heckler, C., Porto, M., ... & Mohile, S. G. (2016) |
| 284 | McAlpine et al. (2015) | Salzer, M. S., Palmer, S. C., Kaplan, K., Brusilovskiy, E., Ten Have, T., Hampshire, M., ... & Coyne, J. C. (2010) |
| 285 | Kang et al. (2018) | Schover, L. R., Canada, A. L., Yuan, Y., Sui, D., Neese, L., Jenkins, R., & Rhodes, M. M. (2012) |
| 286 | Kang et al. (2018) | Schover, L. R., Yuan, Y., Fellman, B. M., Odensky, E., Lewis, P. E., & Martinetti, P. (2013) |
| 287 | Kaltenbaugh et al. (2015) | Scott, K., & Beatty, L. (2013) |
| 288 | Ramsey et al. (2020) | Seitz, D. C. M., Knaevelsrud, C., Duran, G., Waadt, S., Loos, S., & Goldbeck, L. (2014) |
| 289 | Ester et al. (2021) | Shang, J., Wenzel, J., Krumm, S., Griffith, K., & Stewart, K. (2012) |
| 290 | Ester et al. (2021) | Short, C. E., Finlay, A., Sanders, I., & Maher, C. (2018) |
| 291 | Dorri et al. (2019) | Short, C. E., Rebar, A., James, E. L., Duncan, M. J., Courneya, K. S., Plotnikoff, R. C., ... & Vandelanotte, C. (2017) |
| 292 | Ester et al. (2021) | Short, C. E., Rebar, A., James, E. L., Duncan, M. J., Courneya, K. S., Plotnikoff, R. C., ... & Vandelanotte, C. (2017) |
| 293 | Haberlin et al. (2018) | Short, C. E., Rebar, A., James, E. L., Duncan, M. J., Courneya, K. S., Plotnikoff, R. C., ... & Vandelanotte, C. (2017) |
| 294 | Wang et al. (2022) | Short, C. E., Rebar, A., James, E. L., Duncan, M. J., Courneya, K. S., Plotnikoff, R. C., ... & Vandelanotte, C. (2017) |
| 295 | Ester et al. (2021) | Singh, B., Spence, R. R., Sandler, C. X., Tanner, J., & Hayes, S. C. (2020) |
| 296 | Zheng et al. (2020) | Smith, S. K., MacDermott, K., Amarasekara, S., Pan, W., Mayer, D., & Hockenberry, M. (2019) |
| 297 | Zheng et al. (2020) | Somers, T. J., Abernethy, A. P., Edmond, S. N., Kelleher, S. A., Wren, A. A., Samsa, G. P., & Keefe, F. J. (2015) |
| 298 | Kamalumpundi et al. (2022) | Somers, T. J., Kelleher, S. A., Westbrook, K. W., Kimmick, G. G., Shelby, R. A., Abernethy, A. P., & Keefe, F. J. (2016) |
| 299 | Zheng et al. (2020) | Somers, T. J., Kelleher, S. A., Westbrook, K. W., Kimmick, G. G., Shelby, R. A., Abernethy, A. P., & Keefe, F. J. (2016) |
| 300 | Ester et al. (2021) | Spark, L. C., Fjeldsoe, B. S., Eakin, E. G., & Reeves, M. M. (2015) |
| 301 | Zheng et al. (2020) | Stinson, J. N., Jibb, L. A., Nguyen, C., Nathan, P. C., Maloney, A. M., Dupuis, L. L., ... & Johnston, D. L. (2015) |
| 302 | Dorri et al. (2019) | Sturgeon, K. M., Dean, L. T., Heroux, M., Kane, J., Bauer, T., Palmer, E., ... & Schmitz, K. (2017) |
| 303 | Haberlin et al. (2018) | Sturgeon, K. M., Dean, L. T., Heroux, M., Kane, J., Bauer, T., Palmer, E., ... & Schmitz, K. (2017) |
| 304 | Zheng et al. (2020) | Sun, Y., Jiang, F., Gu, J. J., Wang, Y. K., Hua, H., Li, J., ... & Ding, G. (2017) |
| 305 | Qan'ir et al. (2019) | Sundberg, K., Wengström, Y., Blomberg, K., Hälleberg-Nyman, M., Frank, C., & Langius-Eklöf, A. (2017) |
| 306 | Buneviciene et al. (2021) | Trinh, L., Arbour-Nicitopoulos, K. P., Sabiston, C. M., Berry, S. R., Loblaw, A., Alibhai, S. M., ... & Faulkner, G. E. (2018) |
| 307 | Ester et al. (2021) | Trinh, L., Arbour-Nicitopoulos, K. P., Sabiston, C. M., Berry, S. R., Loblaw, A., Alibhai, S. M., ... & Faulkner, G. E. (2018) |
| 308 | Buneviciene et al. (2021) | Uhm, K. E., Yoo, J. S., Chung, S. H., Lee, J. D., Lee, I., Kim, J. I., ... & Hwang, J. H. (2017) |
| 309 | Dorri et al. (2019) | Uhm, K. E., Yoo, J. S., Chung, S. H., Lee, J. D., Lee, I., Kim, J. I., ... & Hwang, J. H. (2017) |
| 310 | Ester et al. (2021) | Uhm, K. E., Yoo, J. S., Chung, S. H., Lee, J. D., Lee, I., Kim, J. I., ... & Hwang, J. H. (2017) |
| 311 | Haberlin et al. (2018) | Uhm, K. E., Yoo, J. S., Chung, S. H., Lee, J. D., Lee, I., Kim, J. I., ... & Hwang, J. H. (2017) |
| 312 | Kiss et al. (2019) | Uhm, K. E., Yoo, J. S., Chung, S. H., Lee, J. D., Lee, I., Kim, J. I., ... & Hwang, J. H. (2017) |
| 313 | Buneviciene et al. (2021) | Urech, C., Grossert, A., Alder, J., Scherer, S., Handschin, B., Kasenda, B., ... & Hess, V. (2018) |
| 314 | Kamalumpundi et al. (2022) | Urech, C., Grossert, A., Alder, J., Scherer, S., Handschin, B., Kasenda, B., ... & Hess, V. (2018) |
| 315 | Kiss et al. (2019) | Vallance, J. K., Courneya, K. S., Plotnikoff, R. C., Yasui, Y., & Mackey, J. R. (2007) |
| 316 | Singleton et al. (2022) | Vallance, J. K., Nguyen, N. H., Moore, M. M., Reeves, M. M., Rosenberg, D. E., Boyle, T., ... & Lynch, B. M. (2020) |
| 317 | Kiss et al. (2019) | Valle, C. G., Deal, A. M., & Tate, D. F. (2017) |
| 318 | Wang et al. (2022) | Valle, C. G., Deal, A. M., & Tate, D. F. (2017) |
| 319 | Ester et al. (2021) | Valle, C. G., Tate, D. F., Mayer, D. K., Allicock, M., & Cai, J. (2013) |
| 320 | Hong et al. (2021) | Valle, C. G., Tate, D. F., Mayer, D. K., Allicock, M., & Cai, J. (2013) |
| 321 | Ester et al. (2021) | Van Blarigan, E. L., Chan, H., Van Loon, K., Kenfield, S. A., Chan, J. M., Mitchell, E., ... & Venook, A. P. (2019) |
| 322 | Wang et al. (2022) | Van Blarigan, E. L., Chan, H., Van Loon, K., Kenfield, S. A., Chan, J. M., Mitchell, E., ... & Venook, A. P. (2019) |
| 323 | Wang et al. (2022) | Van Blarigan, E. L., Kenfield, S. A., Chan, J. M., Van Loon, K., Paciorek, A., Zhang, L., ... & Venook, A. P. (2020) |
| 324 | Golita et al. (2019) | van de Wal, M., Thewes, B., Gielissen, M., Speckens, A., & Prins, J. (2017) |
| 325 | Qan'ir et al. (2019) | van de Wal, M., Thewes, B., Gielissen, M., Speckens, A., & Prins, J. (2017) |
| 326 | Wan et al. (2022) | van de Wal, M., Thewes, B., Gielissen, M., Speckens, A., & Prins, J. (2017) |
| 327 | Golita et al. (2019) | van den Berg, S. W., Gielissen, M. F., Custers, J. A., van der Graaf, W. T., Ottevanger, P. B., & Prins, J. B. (2015) |
| 328 | Singleton et al. (2022) | van den Berg, S. W., Gielissen, M. F., Custers, J. A., van der Graaf, W. T., Ottevanger, P. B., & Prins, J. B. (2015) |
| 329 | Zhang et al. (2022) | van den Berg, S. W., Gielissen, M. F., Custers, J. A., van der Graaf, W. T., Ottevanger, P. B., & Prins, J. B. (2015) |
| 330 | McAlpine et al. (2015) | Van Den Brink, J. L., Moorman, P. W., De Boer, M. F., Hop, W. C., Pruyn, J. F., Verwoerd, C. D., & Van Bemmel, J. H. (2007) |
| 331 | Singleton et al. (2022) | Van Der Hout, A., van Uden-Kraan, C. F., Holtmaat, K., Jansen, F., Lissenberg-Witte, B. I., Nieuwenhuijzen, G. A., ... & Verdonck-de Leeuw, I. M. (2020) |
| 332 | Wan et al. (2022) | Van Der Hout, A., van Uden-Kraan, C. F., Holtmaat, K., Jansen, F., Lissenberg-Witte, B. I., Nieuwenhuijzen, G. A., ... & Verdonck-de Leeuw, I. M. (2020) |
| 333 | Singleton et al. (2022) | Ventura, F., Sawatzky, R., Öhlén, J., Karlsson, P., & Koinberg, I. (2017) |
| 334 | Ester et al. (2021) | Villaron, C., Cury, F., Eisinger, F., Cappiello, M. A., & Marqueste, T. (2018) |
| 335 | Singleton et al. (2022) | Visser, A., Prins, J. B., Jansen, L., Radema, S. A., Schlooz, M. S., van Dalen, T., & van Laarhoven, H. W. (2018) |
| 336 | Kim et al. (2019) | Von Ah, D., Carpenter, J. S., Saykin, A., Monahan, P., Wu, J., Yu, M., ... & Unverzagt, F. (2012) |
| 337 | Huang et al. (2019) | Wang DL and Lin M. (2010) |
| 338 | Wan et al. (2022) | Wang, Q. Q., Zhao, J., Huo, X. R., Wu, L., Yang, L. F., Li, J. Y., & Wang, J. (2018) |
| 339 | Ester et al. (2021) | Webb, J., Fife-Schaw, C., & Ogden, J. (2019) |
| 340 | Ester et al. (2021) | Webb, J., Peel, J., Fife-Schaw, C., & Ogden, J. (2019) |
| 341 | Ester et al. (2021) | Weiner, L. S., Takemoto, M., Godbole, S., Nelson, S. H., Natarajan, L., Sears, D. D., & Hartman, S. J. (2019) |
| 342 | Singleton et al. (2022) | White, V., Farrelly, A., Pitcher, M., & Hill, D. (2018) |
| 343 | Seiler et al. (2017) | Willems, R. A., Bolman, C. A., Mesters, I., Kanera, I. M., Beaulen, A. A., & Lechner, L. (2015) |
| 344 | Buneviciene et al. (2021) | Willems, R. A., Bolman, C. A., Mesters, I., Kanera, I. M., Beaulen, A. A., & Lechner, L. (2017) |
| 345 | Huang et al. (2019) | Willems, R. A., Bolman, C. A., Mesters, I., Kanera, I. M., Beaulen, A. A., & Lechner, L. (2017) |
| 346 | Seiler et al. (2017) | Willems, R. A., Bolman, C. A., Mesters, I., Kanera, I. M., Beaulen, A. A., & Lechner, L. (2017) |
| 347 | Golita et al. (2019) | Willems, R. A., Mesters, I., Lechner, L., Kanera, I. M., & Bolman, C. A. (2017) |
| 348 | Ester et al. (2021) | Wilson, D. B., Porter, J. S., Parker, G., & Kilpatrick, J. (2005) |
| 349 | McAlpine et al. (2015) | Winzelberg, A. J., Classen, C., Alpers, G. W., Roberts, H., Koopman, C., Adams, R. E., ... & Taylor, C. B. (2003) |
| 350 | Seiler et al. (2017) | Wolvers, M. D., Bruggeman-Everts, F. Z., Van der Lee, M. L., Van de Schoot, R., & Vollenbroek-Hutten, M. M. (2015) |
| 351 | Kang et al. (2018) | Wootten, A. C., Abbott, J. A. M., Chisholm, K., Austin, D. W., Klein, B., McCabe, M., ... & Costello, A. J. (2014) |
| 352 | Golita et al. (2019) | Wootten, A. C., Abbott, J. A. M., Meyer, D., Chisholm, K., Austin, D. W., Klein, B., ... & Costello, A. J. (2015) |
| 353 | Qan'ir et al. (2019) | Wootten, A. C., Abbott, J. A. M., Meyer, D., Chisholm, K., Austin, D. W., Klein, B., ... & Costello, A. J. (2015) |
| 354 | Wan et al. (2022) | Wright, H., Martin, F., Clyne, W., Clark, C. C., Matouskova, G., McGillion, M., & Turner, A. (2021) |
| 355 | Wan et al. (2022) | Xia, L. (2020) |
| 356 | Kamalumpundi et al. (2022) | Yanez, B., McGinty, H. L., Mohr, D. C., Begale, M. J., Dahn, J. R., Flury, S. C., ... & Penedo, F. J. (2015) |
| 357 | Qan'ir et al. (2019) | Yanez, B., McGinty, H. L., Mohr, D. C., Begale, M. J., Dahn, J. R., Flury, S. C., ... & Penedo, F. J. (2015) |
| 358 | Buneviciene et al. (2021) | Yang, J., Weng, L., Chen, Z., Cai, H., Lin, X., Hu, Z., ... & Liu, M. (2019) |
| 359 | Zheng et al. (2020) | Yang, J., Weng, L., Chen, Z., Cai, H., Lin, X., Hu, Z., ... & Liu, M. (2019) |
| 360 | Wan et al. (2022) | Yin, G., Guo, X., Lv, X., Yu, K., & Liu, C. (2021) |
| 361 | Kiss et al. (2019) | Yun, Y. H., Lee, K. S., Kim, Y. W., Park, S. Y., Lee, E. S., Noh, D. Y., ... & Park, S. (2012) |
| 362 | McAlpine et al. (2015) | Yun, Y. H., Lee, K. S., Kim, Y. W., Park, S. Y., Lee, E. S., Noh, D. Y., ... & Park, S. (2012) |
| 363 | Seiler et al. (2017) | Yun, Y. H., Lee, K. S., Kim, Y. W., Park, S. Y., Lee, E. S., Noh, D. Y., ... & Park, S. (2012) |
| 364 | Zhang et al. (2022) | Yun, Y. H., Lee, K. S., Kim, Y. W., Park, S. Y., Lee, E. S., Noh, D. Y., ... & Park, S. (2012) |
| 365 | Zheng et al. (2020) | Yun, Y. H., Lee, K. S., Kim, Y. W., Park, S. Y., Lee, E. S., Noh, D. Y., ... & Park, S. (2012) |
| 366 | Ester et al. (2021) | Yun, Y. H., Lim, C. I., Lee, E. S., Kim, Y. T., Shin, K. H., Kim, Y. W., ... & Shin, A. (2020) |
| 367 | Singleton et al. (2022) | Zachariae, R., Amidi, A., Damholdt, M. F., Clausen, C. D., Dahlgaard, J., Lord, H., ... & Ritterband, L. M. (2018) |
| 368 | Huang et al. (2019) | Zeng YL and Wang XL. (2008) |
| 369 | Golita et al. (2019) | Zernicke, K. A., Campbell, T. S., Speca, M., McCabe-Ruff, K., Flowers, S., & Carlson, L. E. (2014) |
| 370 | Huang et al. (2019) | Zhang M, Wang WL, Zhao J, et al. (2013) |
| 371 | Ester et al. (2021) | Zhang, X. (2017) |
| 372 | Huang et al. (2019) | Zhao, Y., Yan, D., & Xu, Y. D. (2014) |
| 373 | Huang et al. (2019) | Zhou S and Su FJ. (2017) |
| 374 | Singleton et al. (2022) | Zhou, K., Wang, W., Zhao, W., Li, L., Zhang, M., Guo, P., ... & Li, X. (2020) |
| 375 | Buneviciene et al. (2021) | Zhu, J., Ebert, L., Liu, X., Wei, D., & Chan, S. W. C. (2018) |
| 376 | Kamalumpundi et al. (2022) | Zhu, J., Ebert, L., Liu, X., Wei, D., & Chan, S. W. C. (2018) |
| 377 | Singleton et al. (2022) | Zhu, J., Ebert, L., Liu, X., Wei, D., & Chan, S. W. C. (2018) |
| 378 | Kaltenbaugh et al. (2015) | Zulman, D. M., Schafenacker, A., Barr, K. L., Moore, I. T., Fisher, J., McCurdy, K., ... & Northouse, L. (2012) |
